# A Bis(arenesulfonyl) Peroxide, an Ambient-Stable Oxidant, Is a Strong p‑Dopant for Organic Semiconductors

**DOI:** 10.1021/acs.chemmater.6c00231

**Published:** 2026-07-10

**Authors:** Suman Kuila, Aniruddha Basu, Megan R. Brown, Spencer J. Gilman, Junxiang Zhang, Kevin Singewald, Glenn Millhauser, Chad Risko, John R. Reynolds, Seth R. Marder, Stephen Barlow

**Affiliations:** † 1877 RASEI, University of Colorado Boulder, Boulder, Colorado 80309, United States; ‡ Department of Chemistry & Center for Applied Energy Research (CAER), 4530 University of Kentucky, Lexington, Kentucky 40506, United States; § School of Chemistry and Biochemistry, School of Materials Science and Engineering, Center for Organic Photonics and Electronics, Georgia Tech Polymer Network, 1372 Georgia Institute of Technology, Atlanta, Georgia 30332, United States; ∥ Department of Chemistry and Biochemistry, University of California, Santa Cruz, California 95064, United States; ⊥ Departments of Chemistry and of Chemical and Biological Engineering, University of Colorado Boulder, Boulder, Colorado 80309, United States; # National Laboratory of the Rockies, Chemistry and Nanoscience Center, Golden, Colorado 80401, United States

## Abstract

Strong p-dopants are required to dope high-ionization energy organic semiconductors for a variety of potential applications, but strong, simple one-electron oxidants are typically sensitive to reduction by atmospheric moisture and thus challenging to store or handle. Here we show that bis­(3,5-bis­(trifluoromethyl)­benzenesulfonyl) peroxide—a dimer formed by two highly oxidizing radicals—can function as a water-stable yet powerful oxidant, cleanly reacting with some organic semiconductors to form two radical cations and two 3,5-bis-(trifluoromethyl)­benzenesulfonate anions, although in other cases sulfonylation reactions can also occur. Notably, this peroxide is capable of p-doping the high-ionization energy polymer poly­[(9,9-dioctylfluorene-2,7-diyl)-*alt*-(benzo­[2,1,3]­thiadiazol-4,7-diyl)] (F8BT) to afford electrical conductivities of up to 0.03 S cm^–1^, while its use with electron-rich poly­(3,4-dialkoxythiophene-2,5-diyl) derivatives can afford values up to 100 S cm^–1^. Quantum-chemical calculations reveal the peroxide oxidant behaves in a fashion mirroring that of relatively oxygen-stable, but highly reducing, n-dopants that have been developed based on dimers of organic radicals or organometallic sandwich compounds.

## Graphical abstract

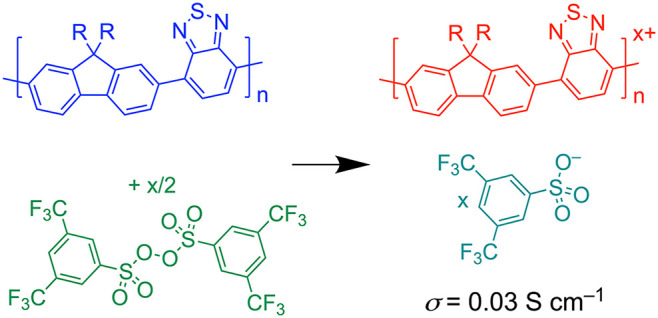

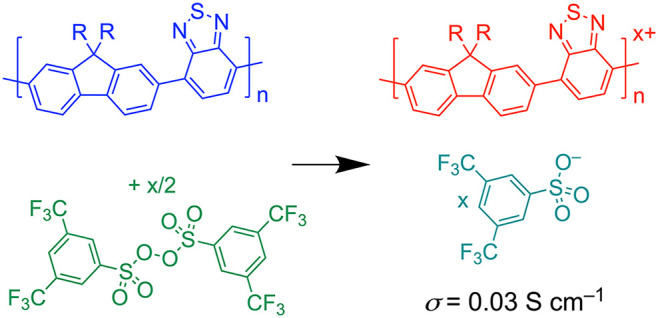

## Introduction

Molecular doping of organic semiconductors (OSCs) with oxidants or reductants can be used to increase conductivity—through filling traps and thus increasing carrier mobility, μ, at low doping levels and/or by increasing free carrier density, *n*, at higher doping levels—and/or to decrease interfacial carrier-injection or extraction barriers^1–6^ in devices including organic^7^ and metal-halide perovskite^8^ light-emitting diodes, organic^9^ and metal-halide perovskite^10^ solar cells, organic field-effect transistors,^11^ and organic thermoelectric generators.^12^ While much work has been done on p-doping, many widely used p-dopants suffer from various drawbacks. For example, the widely used F_4_TCNQ is not a particularly strong oxidant (*E*_red_ = +0.18 V vs FeCp_2_^+/0^;^13^ all literature potentials are quoted relative to this reference, with the caveat that exact values will vary between reports) and also suffers from poor solubility, excessive volatility (which can lead to issues), and facile diffusion within films,^14–17^ while stronger, less volatile cyanocarbon dopants such as F_6_TCNNQ (*E*_red_ = +0.24 V)^13^ and CN_6_–CP (*E*_red_ = +0.78 V)^13^ are even less soluble. Fe^III^ (*e.g*., FeCl_3_,^18^ Fe­(ClO_4_)_3_,^19^ Fe­(CF_3_SO_3_)_3_,^20^ and Fe­(4-MeC_6_H_4_SO_3_)_3_ ^21^) and Ag^I^ salts (*e.g.*, Ag­(CF_3_SO_3_)^22^ and Ag­[N­(SO_2_CF_3_)_2_][Bibr ref23]) oxidize many conjugated polymers, often leading to high levels of conductivity. However, their use may result in the presence of Fe^II^ salts or Ag^0^ in the doped films, leading to uncertainty over exactly what material is being measured and potentially leading to variation in electrical properties depending on the details of how these side products phase segregate from the polymer. In some cases, the counteranions introduced into doped films may be mobile, which can lead to challenges in confining doping to the desired region of a multilayer device.^15^ High-valent metal oxides, notably MoO_3_ and ReO_3_, are vacuum-processable strong p-dopants but exhibit poor miscibilities with OSCs, causing them to aggregate and afford only low extents of doping.^24^ Some strong oxidants are also powerful electrophiles and so can be sensitive to nucleophilic attack by water; for example, NO^+^ (+0.56 to +1.00 V, depending on solvent^24^) reacts with water to form HNO_2_ and H_3_O^+^. A more general problem is that moderate-to-strong p-dopants reacting as simple one-electron oxidants necessarily exhibit highly cathodic reduction potentials and so can undergo one-electron reduction by trace water, especially—as in the case of molybdenum tris­(dithiolene) complexes (+0.12 to +0.39 V)^25^ and CN_6_-CP^13^—in basic solvents, where equilibrium levels of OH^–^ are higher; this precludes dopant storage in, and sometimes even brief exposure to, ambient atmosphere. Accordingly, there is a trade-off between dopant strength and ambient stability for such “simple” dopants. A general strategy to circumvent this trade-off is to identify or develop redox agents—“complex dopants”—in which electron transfer is coupled to other chemical processes.^26^ Complex p-dopant systems to have been studied include: HBr/Me_2_SO mixtures (which presumably lead to the formation of Me_2_SBr^+^Br^–^);^27^ triarylboranes, BAr_3_, some of which act as Lewis acids that complex with Lewis basic OSCs, while others react with water to form Brønsted acids sufficiently strong to protonate OSCs in the initial step of doping;^28,29^ and the tropylium ion, C_7_H_7_^+^, which acts as an electrophile to some OSCs.^30^ In the first of these examples, however, the small and mobile Br^–^ ion is formed, while in the other examples, prediction of reactivity is challenging in that the OSC is thought to participate in reactions (Lewis acid–base pair formation, protonation, electrophilic attack) other than electron-transfer processes. Moreover, the precise reactions and side products are not clear in all cases. There is, therefore, a scarcity of p-dopants that are strong (and thus capable of doping high-IE OSCs such as F8BT) and solution-processable, and that undergo well-defined reactivity with OSCs, even fewer of which can be stored or handled in the presence of water.

One effective class of “complex” n-dopants are the dimers formed by certain highly reducing odd-electron organometallic or organic species; these molecules react to contribute two electrons to the OSC, cleanly forming two monomeric cations, and have been used to n-dope OSCs with *E*_red_ = −2 V and beyond, yet can be handled in air.^31^ We were interested in whether dimeric oxidants, X–X, with reactivity mirroring that of the dimeric n-dopants, *i.e.*, reacting through coupled bond-cleavage and electron-transfer processes to give an overall reaction

1 $$X_{2}+2e^{-}\to {2X}^{-}$$

could be similarly effective p-dopants. Specifically, we hypothesized that these dimers might be strong p-dopants that are relatively water stable (while recognizing that the water stability of successfully doped OSCs would likely depend on the redox potential of the OSC) and that they might react cleanly with OSCs, with the OSC being involved only in “outer sphere” processes (*i.e*., in electron-transfer reactions and not in bond-forming or breaking reactions). The elemental halogens serve as a proof-of-principle but are not practical dopants due to their volatility, their small diffusion-prone ions, the possibility of competing halogenation reactions, and, except for the extremely hazardous F_2_, only mildly oxidizing potentials. The O–O-linked peroxy species benzoyl peroxide, (PhCO_2_)_2_,^32,33^ and potassium persulfate, K_2_S_2_O_8_,^34^ have both been used to p-dope spiro-OMeTAD. More recently, a variety of polymer films were p-doped by immersion in acetonitrile solutions containing persulfate salts and Li^+^[N­(SO_2_CF_3_)_2_]^−^, this reaction proceeding to a much greater extent when catalyzed by a gold substrate.^35^ Although persulfate ion (S_2_O_8_^2–^) is a particularly strong oxidant in a thermodynamic sense and kinetically rather stable to water, it is also rather slow, especially in the absence of a gold catalyst, in reacting with more challenging OSCs.^35^ Moreover, its common salts are poorly soluble, and its reduction product, the sulfate ion, SO_4_^2–^, is fairly small, although this latter issue can be circumvented by its use in conjunction with Li^+^[N­(SO_2_CF_3_)_2_]^−^,^35^ the [N­(SO_2_CF_3_)_2_]^−^ anion being incorporated into the film to balance the holes introduced to the OSC. We were interested in whether neutral bis­(sulfonyl) peroxides, (RSO_3_)_2_ (**1**_**2**_, Chart [Fig chart1]), would serve as organic-soluble analogues of persulfate salts and enable some of these drawbacks to be overcome. **1**_**2**_ derivatives accomplish a range of chemical transformations, including conversion of arenes to arylsulfonylates^36–39^ (which can be further transformed to phenols or fluoroarenes^40^), initiation of radical polymerizations,^36,37^ oxidation of alkyl arenes to benzoic acid derivatives,^41^ disulfonylation of the C=C bond of stilbene,^42^ oxidation of amines to imines,^43^ iodination of arenes,^44^ and the addition of arenesulfonyl and acyloxy groups across the C=C bond of cyclic enol ethers,^45^ but have been little investigated as oxidants for redox-active molecules. Promisingly, however, while simple arenes, ArH, were converted to MeSO_2_OAr by (MeSO_3_)_2_, (**1a**_**2**_), redox-active arenes such as 1,4-dimethoxybenzene (*E*_ox_ = +0.97 V^46^) and thianthrene (*E*_ox_ = +0.90 V^47^) were found to be oxidized to their radical cations instead,^39^ presumably according to eq 2,

2 $${\mathrm{1a}}_{2}+2\mathrm{ArH}\to 2{[\mathrm{ArH}]}^{•+}+2{\mathrm{1a}}^{-}$$

**1 chart1:**
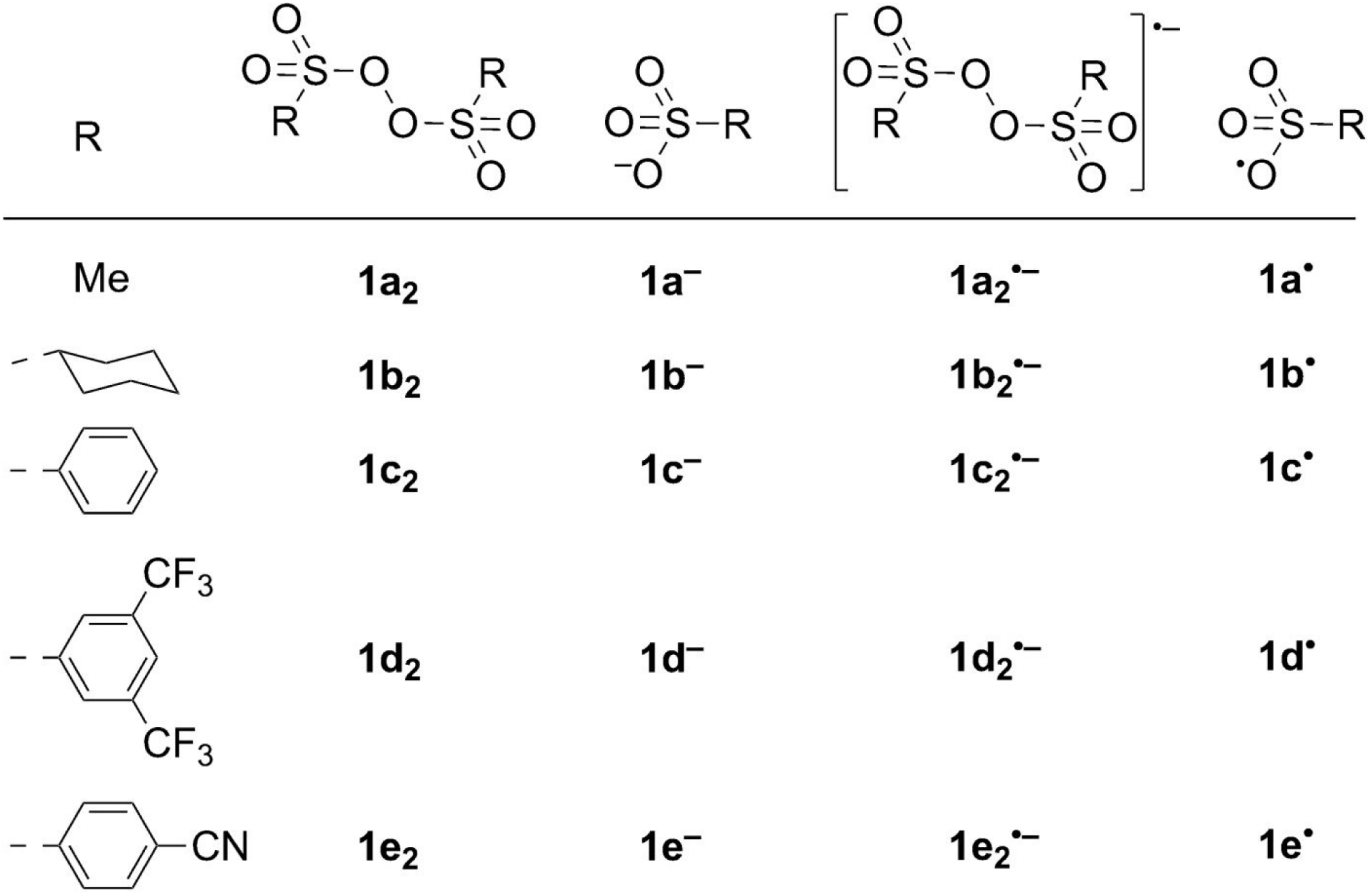
Structures of Bis­(sulfonyl) peroxides (**1_2_**), the Corresponding Sulfonate Anions (**1** ^–^), and Related Species Discussed in this Work

The oxidation potentials of these particular arenes are beyond the reach of all but a few of the p-dopants that have been used to date. On the other hand, **1a**_**2**_ is reported to be prone to explosive decomposition.^37^ Accordingly, we chose to focus on bis­(arenesulfonyl) peroxides. Although some examples, such as (PhSO_2_)_2_, **1c**_**2**_ (Chart [Fig chart1]), are also reported to be capable of violent decomposition,^36,38,42^ such instability has *not* been reported for examples with electron-withdrawing substituents, such as **1d**_**2**_ and **1e**_**2**_ (Chart [Fig chart1]),^38,45^ although it is still suggested that they be handled with care (see Synthesis and Stability section below). In addition, substituted arenesulfonate ions (such as **1d** ^–^ and **1e** ^–^, Chart [Fig chart1]), which are anticipated to be formed in doping reactions according to eq 2, are larger than **1a** ^–^ and thus presumably less prone to diffusion within films or to act as electrostatic traps for charge carriers.

Here, we report on investigations of one of these bis­(arenesulfonyl) peroxides, **1d**_**2**_ (Chart [Fig chart1]), as a p-dopant for OSCs. Specifically, we show it is relatively stable when dissolved in wet organic solvents, yet it is a strong p-dopant, oxidizing a variety of OSC molecules and polymers, including examples with redox potentials as anodic at ca. +1.0 V vs FeCp_2_^+/0^, in solution, and that it can afford substantial electrical conductivities in a variety of OSCs through both codeposition from solution and sequential doping. We also use DFT calculations to explore the analogy between this dimeric oxidant and reductants formed by 19-electron organometallic sandwich compounds or organic radicals.

## Experimental Section

### Synthesis and Characterization

Dopants **1d**_**2**_ and **1e**_**2**_ were synthesized based on literature procedures^45^ (see Supporting Information Section 1.1–1.3 for more details, including safety precautions, and Figures S1–S5 for NMR spectra). NBu_4_^+^ **1d** ^–^ was also synthesized following the literature^48^ (see Supporting Information Section 1.4 and Figures S6–S9). Magic Blue and Magic Green were obtained from Sigma-Aldrich and synthesized according to the literature,^49,50^ respectively. rr-P3HT (90% regioregular, electronic grade, average molecular weight 50000–70000 g mol^–1^) and F8BT (*M*_w_ = 13000 g mol^–1^, PDI = 1.35) were obtained from Reike Metals and Lumtec, respectively. Spiro-OMeTAD and TAPC were purchased from Sigma-Aldrich and 1-Materials, respectively, and used without further purification. PE_2_ (batch *su*)^20^ and EPE-1.5E^51^ were prepared as previously reported. mCP-tBu was synthesized according to a modification of a literature procedure (see Supporting Information Section 1.5 and Figures S10–S11).^52^ ^1^H, ^19^F­{^1^H}, and ^13^C­{^1^H} NMR spectra were recorded with a Bruker 400 MHz NMR spectrometer. Spectra were processed using MestReNova and referenced to tetramethylsilane (TMS) (δ_H_ = 0 ppm; δ_C_ = 0 ppm) using the residual protonated solvent signals or the carbon solvent shift as appropriate. Elemental analyses were obtained from Atlantic Microlab. Thermogravimetric analysis data for **1d**_**2**_ and **1e**_**2**_ (Supporting Information, Figure S12) were acquired in air using a TA Instruments Discovery TGA 500 instrument with a scan rate of 20 °C min^–1^. Optical absorption spectra were recorded in transmission mode using a Cary 5000 spectrophotometer. All solution UV–vis spectra were collected in 1 cm path length quartz cuvettes. All chemical doping experiments were performed in anhydrous dichloromethane. Stock solutions were prepared inside the glovebox, and a measured volume of the sample solution was transferred to a screw-cap cuvette. Oxidant stock solution was then gradually titrated into the sample solution inside the glovebox. Blank spectra were recorded using the pure solvent.

### Solution Investigations of Reactivity

To investigate the counterion formed on **1d**_**2**_ doping, spiro-OMeTAD or TAPC were mixed with 0.5 eq. **1d**_**2**_ in CD_2_Cl_2_ under nitrogen, stirred for 30 min, and then investigated using ^1^H and ^19^F NMR spectroscopy (see Figures S19–S21 in the Supporting Information). To investigate the possibility of reactions other than the desired electron-transfer reactions, spiro-OMeTAD, TAPC, or mCP-tBu (6.2, 6.3, or 6.3 mg, respectively) were mixed with 0.5 eq. of **1d**_**2**_ (1.5, 2.9, or 2.9 mg, respectively) in dry CH_2_Cl_2_ (10 mL) under nitrogen and stirred for 30 min, during which time the solutions took on the characteristic coloration of the corresponding radical cations. Aqueous Na_2_S_2_O_4_ (6 mL) was then added, resulting in the loss of coloration; the organic layer was extracted with CH_2_Cl_2_, dried on Na_2_SO_4_, filtered, evaporated under reduced pressure, and dissolved in CD_2_Cl_2_ and investigated using ^1^H and ^19^F NMR spectroscopy (see Figures S23–S28 in the Supporting Information). In the case of mCP-tBu, where a strong ^19^F signal is seen, the sample was washed with more water, dried, and reinvestigated. In the case of mCP-tBu, similar experiments were carried out using mass spectrometry, but at ca. 10 × higher concentration (Supporting Information, Figure S29).

### Thin-Film Fabrication and Conductivity Measurements

For PE_2_ and EPE-1.5E: Polymer films were prepared by blade coating onto glass slides. To mitigate air doping, the films were loaded into a glovebox and, in some cases, annealed on a hot plate for 15 min. The films were then allowed to cool to room temperature, and ca. 60 μL of an acetonitrile solution of **1d**_**2**_ (5 mM for PE_2_ and 1 mM for EPE-1.5E) was dispensed onto the film and let sit for 1 min. The dopant solution was removed, and the film was washed with acetonitrile three times. The films were removed from the glovebox and placed under vacuum (10 min) to remove any residual acetonitrile. Resistance measurements were carried out with a Signatone SP4 four-point line probe with a Keithley 2400 SourceMeter. Doped polymer film thickness was then determined using a Bruker DektakXT profilometer. The resistance value and film thickness were then used to calculate conductivity.^53^ Values of 104 ± 50, 25.5 ± 10.4, 27.4 ± 2.3, and 0.22 ± 0.06 S cm^–1^ were obtained for unannealed PE_2_, unannealed EPE-1.5E, 250 °C-annealed EPE-1.5E, and 325 °C-annealed EPE-1.5E, respectively.

For rr-P3HT, spiro-OMeTAD, and F8BT, chromium/gold (Cr/Au) electrodes were thermally evaporated on previously cleaned and UV-Ozone-treated glass substrates at a 0.5 Å s^–1^ deposition rate. The Cr/Au electrode-coated glass substrates were further cleaned and treated with UV-Ozone before OSC film fabrication. For codeposition, solutions of **1d**_**2**_ in chloroform were added to a solution of the OSC in the same solvent at different mol %, so that the final concentration of the doped semiconductor was 10 mg mL^–1^. The doped semiconductor solution was spin-coated onto the Cr/Au electrode-coated glass substrates at 2000 rpm and annealed at 70 °C for 5 min. The current–voltage characteristics of the films were recorded using a Keithley 2400 SourceMeter. In the case of rr-P3HT, dopant-induced aggregation was observed in solution, and the method was not pursued further. In the case of both spiro-OMeTAD and F8BT, the use of 50 mol % dopant was found to give the highest conductivity values (1.3 × 10^–3^ and 1.1 × 10^–3^ S cm^–1^ respectively). For sequential doping, an OSC solution in chlorobenzene (10 mg mL^–1^) was spin-coated onto the Cr/Au electrode-coated glass substrates at 2000 rpm and annealed at 120 °C for 5 min. Dopant solutions in acetonitrile with variable concentration were spin-coated dynamically on OSC films at 2000 rpm and annealed at 70 °C for 5 min before measurement of current–voltage characteristics using the Keithley 2400 SourceMeter. For both P3HT and F8BT, the highest values (1.0 × 10^1^ and 3.0 × 10^–2^ S cm^–1^, respectively) were obtained using a dopant solution with a concentration of 20 mg mL^–1^. Acetonitrile solutions were found to poorly wet spiro-OMeTAD films, so sequential doping of this OSC was not pursued further.

### ESR Measurements

Continuous wave (CW-ESR) experiments were acquired with 5 mM of **1d**_**2**_ dissolved in acetonitrile or, along with 100 mM of 5,5-dimethyl-1-pyrroline-*N*-oxide (DMPO) as a spin trap in a 9:1 mixture of acetonitrile:water. A 70 μL portion of solution was injected into a 1.5 mm OD capillary (Friedrich & Dimmock Borosilicate Capillary). The CW-ESR spectra were recorded using a Bruker ElexSys E500 spectrometer operating at X-band (9.33 GHz), equipped with an ER 4122SHQE resonator and a variable temperature controller. Temperatures were achieved using a liquid-nitrogen finger dewar and gas flow controller and were calibrated using a digital thermometer while the magnetic field was off. The following CW-ESR parameters were used for all experiments: center field = 3330 G; sweep width = 200 G; modulation amplitude = 1 G; modulation frequency = 100 kHz; conversion time = 20.48 ms; and number of scans = 5 or 20. The reported ESR intensity (see Supporting Information Figure S31 and Section 2.4) is equal to the doubly integrated intensity after background and baseline subtraction.

### Quantum-Chemical Calculations

Density functional theory (DFT) and time-dependent DFT (TDDFT) calculations were performed using the Gaussian 16 Rev. A.03 software suite^54^ at the M06-2X^55^/6-311+G­(3df,2p)^56–59^ level of theory. The polarizable continuum model (PCM)^60^ was used to represent dielectric effects in acetonitrile (ε = 35.7) and chloroform (ε = 4.7). The optimized geometries of the **1**_**2**_, **1**_**2**_^**•–**^, **1** ^**•**^, and **1** ^–^ species underwent frequency calculations to confirm the absence of imaginary frequencies and to calculate the Gibbs free energy of the species at 298.15 K. Species that were unable to converge without imaginary frequencies were omitted. TDDFT calculations were performed on the optimized geometries to determine excitation energies and associated oscillator strengths. An artificial broadening of σ = 0.3 was applied to each peak to simulate the optical absorbance spectra. Molecular orbitals (Figure 3) and natural transition orbitals (Supporting Information, Table S4) were visualized using the GaussView 6.1.1 software^61^ package with an isovalue of 0.02.

## Results and Discussion

### Synthesis and Stability

As noted above, bis­(methanesulfonyl) peroxide, **1a**_**2**_, was previously shown to be a rather strong oxidant but can decompose explosively,^37^ while the MeSO_3_^–^ (**1a** ^–^) anion expected to be formed on doping OSCs is likely rather mobile. To examine how bis­(arenesulfonyl) peroxides would compare in terms of oxidant strength to a comparably sized bis­(alkanesulfonyl) peroxide, **1b**_**2**_ (Chart [Fig chart1]), Gibbs free energies, Δ*G*, for the reaction

3 $$0.5{\left({\mathrm{RSO}}_{3}\right)}_{2}+e^{-}\to {{\mathrm{RSO}}_{3}}^{-}$$

in acetonitrile were obtained from density functional theory (DFT) calculations (at the M06-2X/6-311+G­(3df,2p) level,^55–59^ which has previously been used in other studies of peroxide species;^62^ see below and Supporting Information, Tables S1–S2, for more details). The calculations suggest that replacing the cyclohexyl groups of **1b**_**2**_ with phenyl groups in **1c**_**2**_ leads to a slightly stronger oxidant; Δ*G*(0.5**1**_**2** _+ e^–^ → **1** ^–^) for **1c**_**2**_ is 2.0 kcal mol^–1^ (0.087 eV) more exergonic than for **1b**_**2**_. However, it should be reiterated that there are reports of the violent decomposition of **1c**_**2**_,^36,38,42^ as in the case of **1a**_**2**_. Incorporation of electron-withdrawing groups on the aryl ring, which is experimentally shown to lead to more stable bis­(arenesulfonyl) peroxides,^38,45^ is calculated to further increase the effective oxidant strength. We chose to synthesize two examples that have recently been reported in the literature:^45^ Δ*G*(0.5**1**_**2**_ + e^–^ → **1** ^–^) values for derivatives with R = 3,5-bis­(trifluoromethyl)­phenyl (**1d**_**2**_) and R = 4-cyanophenyl (**1e**_**2**_) are calculated to be more exergonic than that for their R = cyclohexyl analogue (**1b**_**2**_) by 7.6 and 6.1 kcal mol^–1^ (0.33 and 0.26 eV), respectively. Both **1d**_**2**_ and **1e**_**2**_ were obtained in one straightforward step involving the reaction of the commercially available RSO_2_Cl derivative with aqueous hydrogen peroxide according to ref 45; in the case of **1d**_**2**_, we have carried out the reaction on scales of up to 10 g (see Experimental Section and Supporting Information Sections 1.1–1.3). We observed no indications of dangerous room-temperature reactivity for either **1d**_**2**_ or **1e**_**2**_, but thermogravimetric analysis (TGA, Supporting Information, Figure S12) suggested that **1e**_**2**_ decomposes violently at 100 °C, whereas **1d**_**2**_ loses weight more gradually at temperatures from around 120 °C and so, out of an abundance of caution, we focused on **1d**_**2**_ for further investigations. We also avoided further heating of **1d**_**2**_ *except* in dilute solution to 80 °C for electron spin-resonance experiments (see Experimental Section and Supporting Information, Section 2.5) and in some doped films, which were annealed at 70 °C (see Experimental Section). We also avoided using metal spatulas with **1d**_**2**_ and used an antistatic gun during weighing (as recommended in the Supporting Information of ref 45).

**1d**_**2**_ can be stored as a solid in the presence of air for at least a month without any decomposition as detected by ^1^H and ^19^F NMR spectroscopy, while it is soluble in and stable to a variety of solvents, including chloroform (in contrast to the reported reactivity of less electron-poor bis­(arenesulfonyl) peroxides^63^), dichloromethane, and acetonitrile. We avoided aromatic solvents based on the reported reactivity of some arenes with some **1**_**2**_ derivatives.^36–39^

As noted in the introduction, strong, simple one-electron oxidants are generally reduced by trace amounts of water. This can lead to their degradation on storage, which can reduce their effectiveness as dopants and, in some cases, lead to unwanted products that might be incorporated into doped films. To examine if the dimeric character of **1d**_**2**_ helps circumvent this issue, we compared the water stability of **1d**_**2**_ to that of two strong one-electron oxidants—Magic Blue (MB, [(4-BrC_6_H_4_)_3_N]^•+^SbCl_6_^–^, *E*_red_ = +0.70 V^64^) and Magic Green (MG, [(2,4-Br_2_C_6_H_3_)_3_N]^•+^SbCl_6_^–^, *E*_red_ = +1.14 V^64^)—in acetonitrile. As shown in Figure 1, addition of ca. 3 equiv of water to either of these triarylamine radical-cation salts leads to a significant decrease in absorbance, whereas the absorption spectrum of **1d**_**2**_ is unchanged under comparable conditions. We note that experimental data for **1d**_**2**_ and NBu_4_^+^ **1d** ^–^,^48,65,66^ a **1d** ^–^ salt soluble in some of the same low-polarity solvents as **1d**_**2**_ (Supporting Information, Figure S13), and TD-DFT calculations (Supporting Information, Figures S33–34 and Tables S4 and S5) indicate that the electronic spectra of **1d**_**2**_ and **1d** ^–^ are clearly distinguishable from one another. This water tolerance is consistent with the synthesis of **1d**_**2**_ being carried out in the presence of water and is presumably related to kinetic barriers associated with the bond-breaking and electron-transfer processes needed to reduce **1d**_**2**_. This behavior mirrors the relative oxygen stability of some of the strongly reducing dimeric species that we have previously reported.^31^ This kinetic stability to water may help simplify storage and handling and may, in the case of less challenging OSCs, allow for the doping process to be carried out in ambient conditions. In spite of this and the water stability of the monomeric counterion expected to be formed on doping by analogy to eq 2 (**1d** ^–^)^48,65,66^ (as is typical for RSO_3_^–^ anions and also the SbCl_6_^–^ anion from MB or MG), we caution that the cationic OSC species (radical cations, *i.e*., polarons, and bipolarons) formed by higher-IE OSCs on doping will likely still be highly sensitive to reduction by ambient water (as indeed we find for **1d**_2_-doped F8BT).

**Figure 1:**
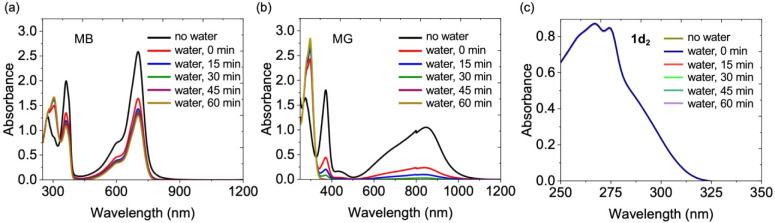
UV–vis-NIR absorption spectra for (a) 0.1 mM Magic Blue (MB), (b) 0.1 mM Magic Green (MG), and (c) 1 mM **1d_2_** solutions in acetonitrile at various times after the addition of water (20 μL water for MG and MB, 200 μL water for **1d_2_**; in all cases, in 3 mL solution, water concentrations were ca. 0.3 mM for MG and MB and 3 mM for **1d_2_**, *i.e.*, ca. 3× the dopant concentration in each case) in a 1 cm cuvette. The higher concentrations of both oxidant and water used for **1d_2_** were used due to the much lower absorptivity of this species relative to MB and MG.

### Solution Oxidant Behavior

We examined the reaction of **1d**_**2**_ with a variety of molecular and polymeric species (see Experimental Section for sources of materials) in dry dichloromethane using UV–vis-NIR spectroscopy (Figure 2, Supporting Information Figure S14–S19). As shown in Figure S14 in the Supporting Information, ferrocene (*E*_ox_ = 0 V vs FeCp_2_^+/0^ by definition) is oxidized to the ferrocenium ion by **1d**_**2**_, as evidenced by the appearance of a feature at around 620 nm, consistent with the previously reported reactivity of **1a**_**2**_ and ferrocene.^39^ Furthermore, taking into account the absorptivity of the ferrocenium ion, the absorbance of the ferrocenium peak is consistent with *all* the ferrocene being oxidized by 0.5 eq. **1d**_**2**_, while approximately the same ferrocenium absorbance is obtained if 1 equiv. **1d**_**2**_ is used, or when 1 equiv. MB—a one-electron oxidant—is used in place of **1d**_**2**_; these observations indicate that, as expected, **1d**_**2**_ behaves as an overall two-electron oxidant, *i.e.*, as represented in eqs 1, 2, or 3. Turning to OSCs, **1d**_**2**_ also oxidizes a range of molecular and polymeric species (see Chart [Fig chart2] for chemical structures) in dichloromethane solution, as indicated by the absorption spectra shown in Figure 2, which closely resemble the published radical-cation (polaron) spectra for these OSCs or similar model compounds (see refs 67, 68,69, and 70 for the oxidation products of P3HT, F8BT, spiro-OMeTAD, and TAPC respectively, and ref 71 for the oxidation product of a monomeric model for mCP-tBu). Additional UV–vis-NIR spectra are shown in the Supporting Information for solutions employing different concentrations (Figures S14–S19) and for solid films (Figures S16–S17), and indicate that higher levels of the dopant can lead to higher oxidation levels in some cases, notably to the formation of P3HT singlet bipolarons^67^ and spiro-OMeTAD^n+^ (n = 3, 4)^69^ (see Figures S15 and S17 and associated discussion in the captions). Furthermore, comparison of ^1^H NMR spectra of **1d**_**2**_,^45^ NBu_4_^+^ **1d** ^–^,^48,65,66^ and the reaction mixtures of spiro-OMeTAD and TAPC with **1d**_**2,**_ (Figures S20 and S21; see also Figure S22 for ^19^F NMR data) suggests that the anion formed in the solution redox reaction is indeed **1d** ^–^, consistent with eqs 1–3.

**2 chart2:**
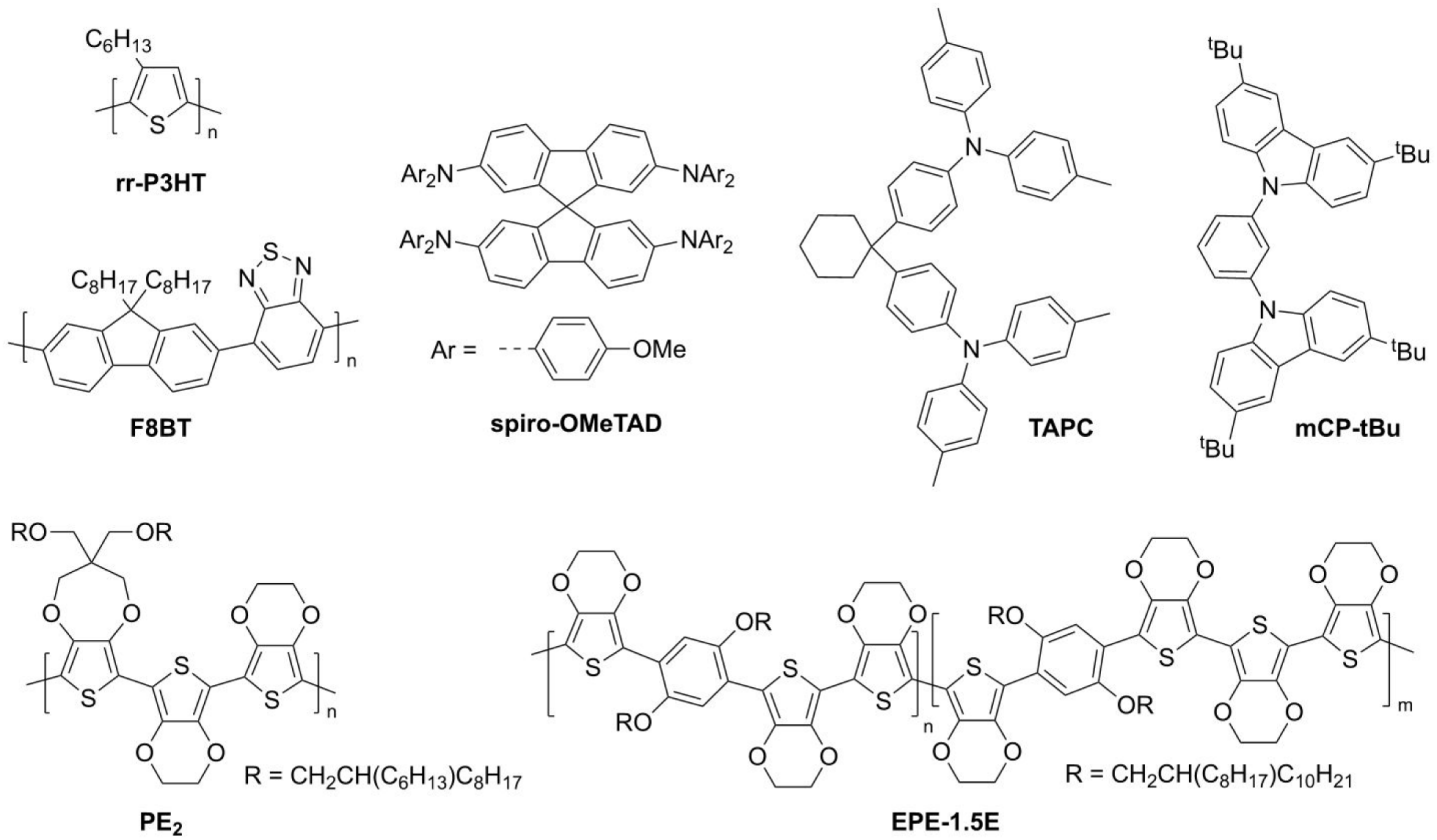
Structures of OSCs Used to Test the Solution Reactivity and Doping Abilities of **1d_2_**

**Figure 2:**
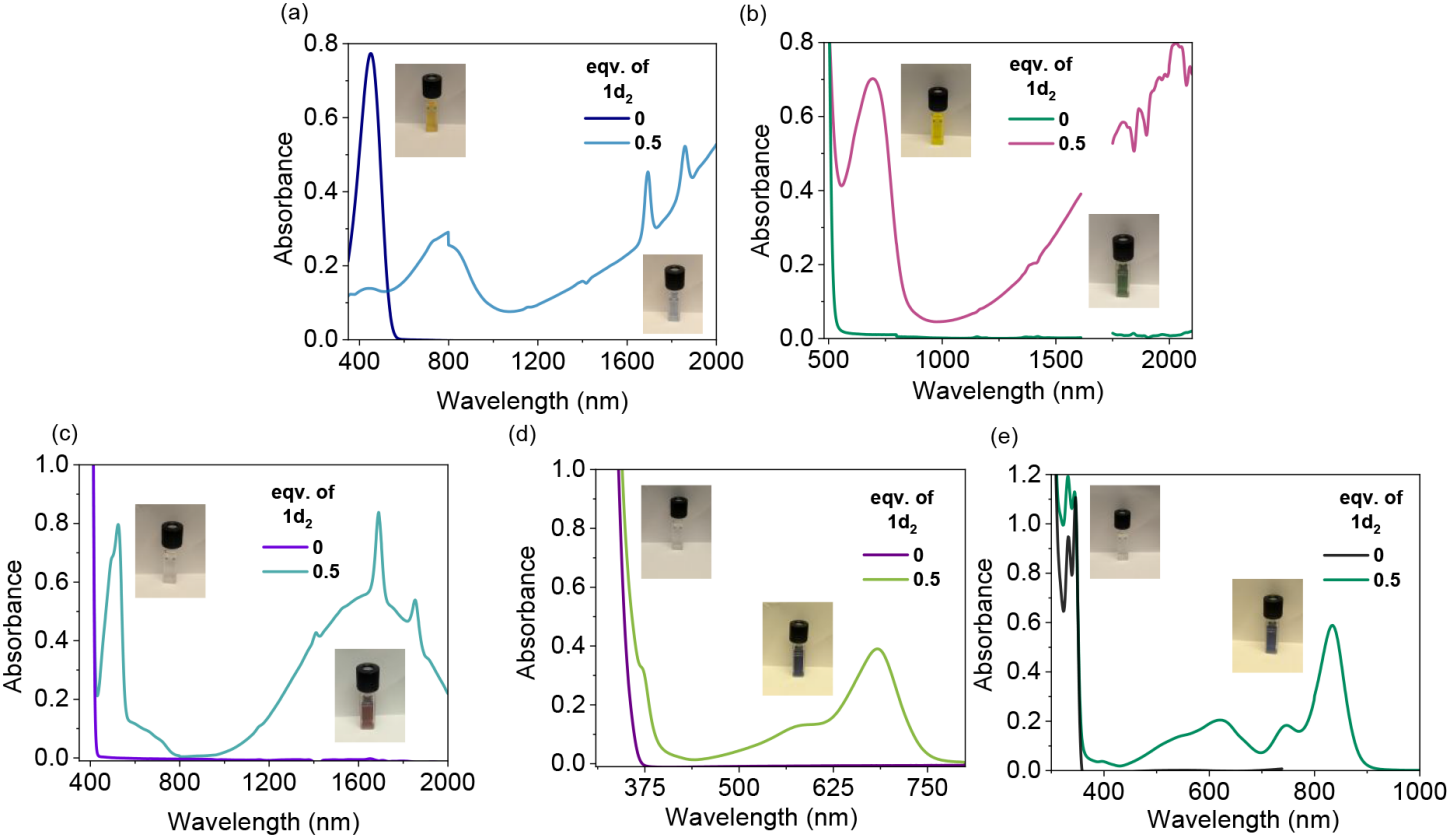
Optical absorption spectra of (a) 0.1 mM rr-P3HT, (b) 1 mM F8BT (a portion of the spectra at ca. 1700 nm has been masked off due to a strong vibronic feature), (c) 0.1 mM spiro-OMeTAD, (d) 0.1 mM TAPC, and (e) 0.1 mM mCP-tBu in dry dichloromethane and their reaction products with 0.5 eq. **1d_2_** under inert atmosphere. Insets show cuvettes of the relevant solutions.

rr-P3HT and spiro-OMeTAD have oxidation potentials close to that of ferrocene (ca. 0.0 to −0.2 V^72–75^ and +0.01 V,^69^ respectively) and thus can be—and have been—doped by a wide variety of oxidants, including relatively weak and stable examples such as F_4_TCNQ and its derivatives,^76–78^ molybdenum tris­(dithiolene) complexes,^79,80^ and O_2_/Li­[N­(SO_2_CF_3_)_2_].^81^ However, the other OSCs are more challenging; the OLED materials TAPC and mCP-tBu are oxidized at +0.61 ^82^ and +0.82 V,^52^ respectively, while F8BT is oxidized at ca. +0.9 to +1.1 V.^75,83,84^ Indeed, only a few oxidants have previously been successfully used to induce positive polarons in F8BT: thianthrene radical cation or NO^+^ in solution,^60^ and [(2,4,6-Me_3_C_6_H_2_)_2_B]^+^[B­(C_6_F_5_)_4_]^−^ ^85^ or F_6_TCNNQ·B­(C_6_F_5_)_3_ in films.^86^ It is worth noting, however, that while **1d**_**2**_ is stable, the **1d**_**2**_-oxidized solutions of high-*E*_ox_ organics such as F8BT are highly sensitive to reduction on exposure to air, presumably by atmospheric moisture.

As noted in the introduction, **1d**_**2**_ derivatives can react with aromatic rings in a variety of ways; thus, if any of these reactions are competitive with the desired outer-sphere reactivity according to eqs 1–3, side products could potentially be formed in doping reactions. To test this possibility, we rereduced reaction mixtures of **1d**_**2**_ and molecular OSCs using aqueous Na_2_S_2_O_4_ (see Experimental Section and Supporting Information, Section 2.3 including Figures S23–S29, for details) and investigated the resulting CH_2_Cl_2_-soluble material using NMR spectroscopy. In the case of spiro-OMeTAD and TAPC, the ^1^H NMR spectra showed only the neutral OSCs with no sign of any side products. In the case of mCP-tBu, the principal recovered product is also the neutral OSC, but ^1^H NMR spectra also showed small quantities of new species (Figure S27). Mass spectra are consistent with the formation of an ArSO_2_O-substituted derivative of mCP-tBu, likely as a mixture of regioisomers, consistent with reactions of arenes according to

4 $${\left({\mathrm{RSO}}_{3}\right)}_{2}+\mathrm{ArH}\to {\mathrm{ArOSO}}_{2}R+{\mathrm{RSO}}_{3}H$$

as reported in the literature.^36–39^ This reaction is presumably sufficiently competitive with doping to be seen in the case of mCP-tBu due to its high oxidation potential, which is anticipated to slow one-electron oxidation, at least via the “electron-transfer first” mechanism (see below), relative to that for spiro-OMeTAD and TAPC.

### Electrical Conductivity of Doped Films

We measured the in-plane electrical conductivity of films of various OSCs (see Chart [Fig chart2] for chemical structures) doped with **1d**_**2**_ (Table 1, Figure S14). Doped films were prepared by solution doping in chloroform followed by spin-casting (“codeposition”), or by exposing spin- or blade-coated polymer films to acetonitrile solutions of **1**_**2**_ (“sequential doping”); the corresponding electrical conductivity values for the doping conditions giving the highest conductivities are summarized in Table 1. Sequential doping of the electron-rich polymers PE_2_ ^87^ and EPE-1.5E^51^ affords conductivity values similar to those obtained with Fe^III^ salts. The highest electrical conductivities we obtained here with P3HT are over an order of magnitude lower than the highest values reported in the literature,^88^ but still compare well to those in many studies. The electrical conductivity obtained in spiro-OMeTAD exceeds that typically obtained with O_2_/Li^+^[N­(SO_2_CF_3_)_2_]^−^ (up to 3 × 10^–5^ S cm^–1^ in ref 81), is more comparable to that obtained with a molybdenum tris­(dithiolene) derivative (ca. 4 × 10^–4^ S cm^–1^),^80^ and falls short of the highest values reported using (PhCO_2_)_2_ (2.4 × 10^–2^ S cm^–1^).^32^ Although doping can also both increase and decrease charge-carrier mobility depending on the interplay of trap filling and effects on OSC packing, these conductivity values, along with the optical spectra indicating radical-cation formation noted in the previous section, are consistent with the conductivity enhancements largely resulting from a dopant-induced increase in charge-carrier density. Of particular interest is the p-doping of F8BT, which has the highest solution oxidation potential (+0.9 to +1.1 V^75,83,84^ ) and solid-state ionization energy (5.8–5.9 eV according to UV photoelectron spectroscopy^89–91^ ) of the materials examined. There are few previous measurements of conductivity in p-doped F8BT. The highest values reported to date are ca. 6 × 10^–6^ S cm^–1^ using either Au-activated (Na^+^)_2_S_2_O_8_^2–^/Li^+^[N­(SO_2_CF_3_)_2_]^−^ or “Magic Blue”,^35^ where, however, there are no obvious polaron peaks in the absorption spectra of the doped films. The much higher polaron concentrations—*i.e*., greater extents of redox reaction, resulting from a stronger oxidizing power—obtained using **1d**_**2**_ (see Figure S16) and thus, likely higher free carrier concentrations, are likely primarily responsible for the much higher conductivities seen in the present study. On the other hand, [(2,4,6-Me_3_C_6_H_2_)_2_B]^+^[B­(C_6_F_5_)_4_]^−^ gives polaron concentrations comparable to those obtained using solution doping of **1d**_**2**_, yet the conductivity is very low (ca. 10^–9^ S cm^–1^);^85^ in this case, it may be that the effect of the additional carriers is more than outweighed by the adverse impact of the bulky [B­(C_6_F_5_)_4_]^−^ anions on the polymer structure and, thus, the average carrier mobility.

**Table 1:** Electrical Conductivities for Organic Semiconductors p-Doped with **1d_2_** ^a^

		Mean Conductivity/S cm^–1^	
OSC	*E*_ox_ vs FeCp_2_^+/0^/V	Codeposition	Sequential Doping
PE_2_	–0.5 ^87^	–	1.0 × 10^2^
EPE-1.5E	–0.2 ^51^	–	3.6 × 10^1^
rr-P3HT	0.0 to −0.2 V^72–75^	–	1.0 × 10^1^
spiro-OMeTAD	0.0 ^69^	1.3 × 10^–3^	–
F8BT	+0.9 to +1.1 ^75,83,84^	1.1 × 10^–3^	3.0 × 10^–2^

a See experimental section for the doping conditions that were used to obtain these values.

### Computational Insights into Reactivity

Various characteristics of **1d**_**2**_, along with a variety of other real and hypothetical **1**_**2**_ derivatives (see Supporting Information, Section 3), were investigated using M06-2X/6-311+G­(3df,2p) DFT calculations to examine the extent to which this dimeric oxidant resembles the dimeric reductants we have previously worked with and to assess the feasibility of different mechanisms possible for the doping reaction. The homolytic bond dissociation energy calculated for neutral **1d**_**2**_ (34.4 kcal mol^–1^ in MeCN) is similar to, but somewhat lower than, values reported for other peroxy species at the same computational level, including hydrogen peroxide (49.7 kcal mol^–1^) and benzoyl peroxide (41.4 kcal mol^–1^).^62^ The corresponding free energy of dissociation, Δ*G*_diss_, for **1d**_**2**_ is 17.9 kcal mol^–1^. This value is comparable to DFT values for some of the more weakly bound dimeric n-dopants that we have examined, such as (N-DMBI)_2_ (Δ*G*_diss_ = +20 kcal mol^–1^),^92^ which is relevant in the context of reaction mechanisms (see below). We attempted to obtain an experimental estimate for the dissociation constant for **1d**_**2**_ using electron spin resonance spectroscopy following previous work on (DMBI)_2_ derivatives^93^ but were unable to observe a signal, suggesting Δ*G*_diss_ > ca. 16 kcal mol^–1^ at room temperature (see Supporting Information, Section 2.5 for more details, as well as for associated discussion of the spin-trapping experiment shown in Figure S31).

In the dimeric n-dopants, one-electron oxidation of the dimers is calculated to result in a considerable lengthening and weakening of the central C–C bonds;^93,94^ this is consistent with the molecular orbital structure in which there is a C–C σ-bonding component to the HOMO of the neutral dimer. In the present systems (Figure S32, Tables S1–S3), one-electron reduction results in an analogous lengthening (from 1.408 to 2.089 Å for **1d**_**2**_) and weakening (Δ*G*_diss_ decreasing from 17.9 to −8.3 kcal mol^–1^) of the O–O bond, which is consistent with an O–O σ-antibonding component seen in the LUMO of the neutral species (Figure 3). In the dimeric n-dopants, DFT calculations and electrochemical experiments^93,94^ suggest that neutral dimers exhibit ionization energies that are 1 eV or more higher than those of the corresponding monomeric radicals, consistent with their moderate stability to air. This situation is mirrored for **1d**_**2**_, the DFT electron affinity of which is over 1 eV smaller than that of the corresponding monomeric radical (Tables S1–S2).

**Figure 3:**
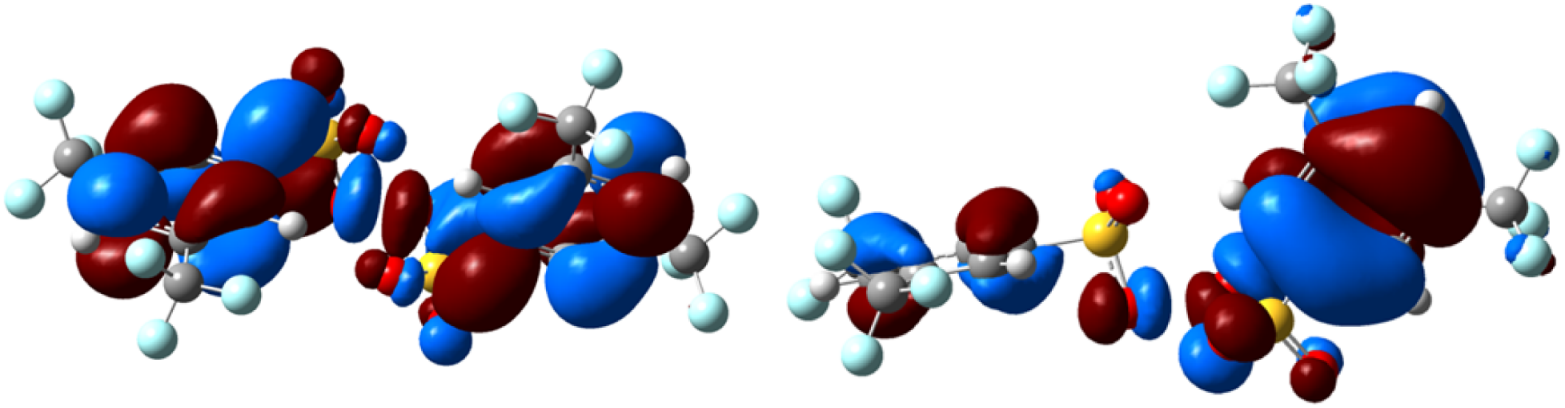
LUMO of **1d_2_** showing O–O antibonding character (left) and α-HOMO for **1d_2_^•–^** (right) from M06-2X/6-311+G­(3df,2p) DFT calculations.

Two doping mechanisms are well established for dimeric reductants: in the “electron-transfer first” mechanism, the dimer transfers an electron to the OSC, with the dimer radical cation subsequently cleaving to afford a closed-shell monomer cation and an odd-electron monomer, which then transfers a second electron, while the “cleavage first” mechanism is viable for more weakly bound dimers, which are in equilibrium with the highly reducing odd-electron monomers that can then serve as one-electron reductants.^31^ Given the close relationship noted above between the dimer/monomer energetic relationships in the dimeric oxidant and the dimeric reductant systems, we anticipate that **1**_**2**_ dopants are likely to react via analogous pathways. First, in the “electron-transfer first” mechanism, **1**_**2**_^**•–**^ cleaves to **1** ^–^ and **1** ^**•**^ (similar to what is proposed in the doping of spiro-OMeTAD by (PhCO_2_)_2_ ^32^) with the latter species subsequently accepting an electron from a second molecule of the OSC, *i.e*.,

5a $$1_{2}+\mathrm{OSC}\to {\mathrm{OSC}}^{•+}+{1_{2}}^{•-}$$

$${1_{2}}^{•-}\to 1^{-}+1^{•}$$ 5b

$$1^{•}+\mathrm{OSC}\to {\mathrm{OSC}}^{•+}+1^{-}$$ 5c

Alternatively, in the “cleavage first” pathway, initial dissociation of the dimer (which has been invoked in other **1**_**2**_ reactivity, such as the initiation of radical polymerizations^36,37^ ) affords two **1** ^**•**^ monomers, which then act as electron acceptors, *i.e.*,

$$1_{2}\to 21^{•}$$ 6a

$$1^{•}+\mathrm{OSC}\to {\mathrm{OSC}}^{•+}+1^{-}$$ 6b

The relative free energies of reactants, intermediates, and products in acetonitrile according to the DFT calculations are shown in Figure 4 for both pathways in the case of doping both a relatively challenging OSC (left), whose doping is only marginally exergonic, and a more easily ionized material (right) using **1d**_**2**_. With the caveat that the activation barriers, Δ*G* ^‡^, for the first steps for each pathway—which are likely to be rate-determining for either pathway—might not directly track with the free-energy changes, Δ*G* ^0^ for those steps, the energies of the intermediates suggest it is likely that the “electron-transfer first” mechanism will dominate for relatively easily ionized OSCs, but that both may be important for materials of borderline thermodynamic feasibility. Thus, the reactivity of **1d**_**2**_ likely mirrors that of the more weakly bound dimeric n-dopants, such as (N-DMBI)_2_, which mechanistic data suggest reacts via both possible pathways.^95^

**Figure 4:**
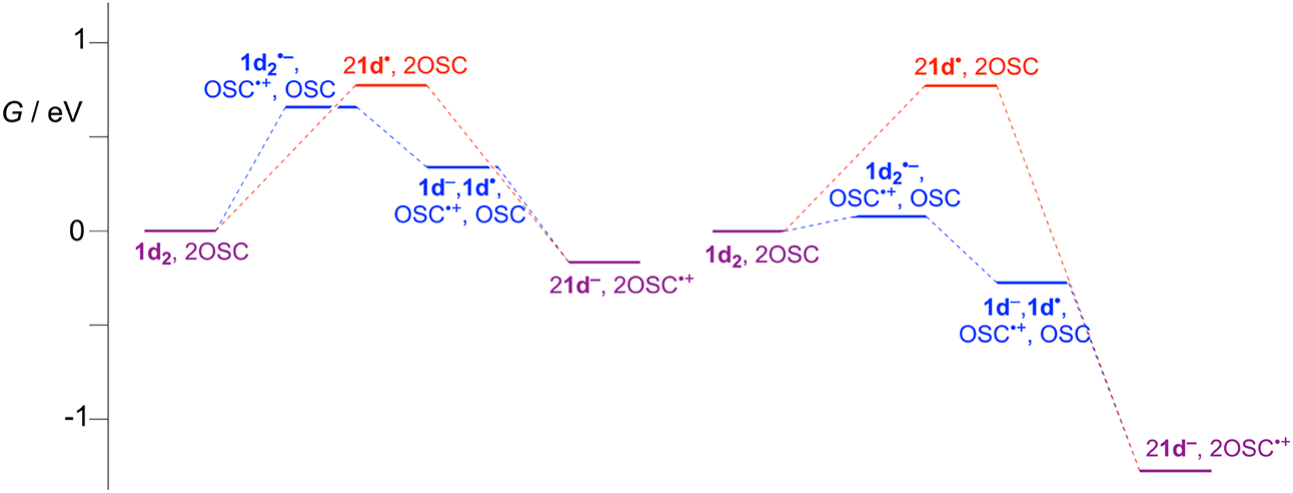
Free energies of reactants, intermediates, and products at 298.15 K for the reactions in MeCN of **1d_2_** with (left) an OSC with relatively high ionization energy (Δ*G*(OSC → OSC^•+^ + e^–^) = 6.7 eV) and thus an only slightly exergonic overall reaction and (right) an OSC with a lower ionization energy (Δ*G*(OSC → OSC^•+^ + e^–^) = 6.2 eV) and more exergonic overall reaction using values of Δ*G*(**1d_2_** → 2**1d^•^**), Δ*G*(**1d_2_^•–^** → **1d** ^–^ + **1d^•^**), Δ*G*(**1d_2_** + *e*− → **1d_2_^•–^**), and Δ*G*(**1d^•^** + *e*− → **1d** ^–^) from DFT (M06-2X/6-311+G­(3df,2p)) calculations. Intermediates for the “electron-transfer first” and “cleavage first” pathways are shown in blue and red, respectively.

Finally, our calculations give some insight into the potential impact of the R group in **1**_**2**_ derivatives on both their thermodynamic doping strength, gauged by Δ*G* for eq 3 (see above), and their likely reactivity via the two pathways noted above (Equation 5 and Equation 6b). Regardless of reaction mechanism,

$$\Delta G\left(0.51_{2}+e^{-}\to 1^{-}\right)=0.5\Delta G\left(1_{2}\to 21^{•}\right)+\Delta G\left(1^{•}+e^{-}\to 1^{-}\right)$$ 7

*i.e.* the dopant strength depends on both the free energy of dissociation of the dimer *and* the acceptor strength of the **1** ^**•**^ radical. Calculations for a range of different examples (Tables S1–S2) show no clear correlation between the free energy of dissociation, Δ*G*(**1**_**2**_ → 2**1** ^**•**^), and the chemical structure (*i.e*., whether R is alkyl or aryl, or, in the aryl examples, the nature of the substituents). The **1** ^**•**^ radicals are more oxidizing, *i.e.*, values of Δ*G*(**1** ^**•**^ *e* ^−^ → **1** ^–^) are more exergonic, for aryl examples with electron-withdrawing substituents (such as **1d**_**2**_ and **1e**_**2**_), as one would expect, although the variation in values between cyclohexyl, phenyl, and different alkyl-substituted phenyl examples is not straightforward. Some of the less straightforward variations in Δ*G*(**1**_**2**_ → 2**1** ^**•**^) and Δ*G*(**1** ^**•**^ → **1** ^–^) cancel when Δ*G*(0.5**1**_**2**_ + e^–^ → **1** ^–^) is calculated according to eq 7. Overall, dopants with R = aryl are thermodynamically stronger, *i.e*., Δ*G*(0.5**1**_**2**_ + e^–^ → **1** ^–^) is more exergonic, than those with R = cyclohexyl (**1b**_**2**_). Furthermore, increasing numbers or strength of electron-withdrawing groups on R = aryl lead to even stronger dopants.

In terms of reaction pathways, the free energy of dissociation, Δ*G*(**1**_**2**_ → 2**1** ^**•**^) is relevant to the feasibility of the “cleavage first” pathway, but the nonstraightforward variation in this quantity makes the influence of structure on the importance of this pathway hard to predict beyond the set of compounds shown in Figure S32 and Tables S1–S2. The ease of reduction of the dimers to their radical anions, Δ*G*(**1**_**2** _+ e^–^ → **1**_**2**_^**•–**^) relates to the feasibility of the “electron-transfer first” pathway: these values are similar for R = cyclohexyl, Ph, and alkylated aryl derivatives, but, as one might expect, become increasingly exergonic with the number and strength of electron-withdrawing substituents on aryl R groups. Thus, the use of electron-withdrawing groups to increase dopant strength will generally also increase reactivity with OSCs. Of course, other practical considerations for future work might include: synthetic feasibility, including availability of appropriate starting materials; solubility; thermal (in)­stability; and substituent size.

## Conclusion

Bis­(3,5-bis­(trifluoromethyl)­benzenesulfonyl), **1d**_**2**_, is relatively stable to water yet a powerful oxidant. It oxidizes a range of redox-active materials in chloroalkane solution to their radical cations, notably F8BT, which has a high oxidation potential of ca. +1 V vs FeCp_2_^+/0^, with the formation of the corresponding 3,5-bis­(trifluoromethyl)­benzenesulfonate, **1d** ^–^, counterion. For some molecules this is rather a clean reaction; however, for at least one example of a less easily oxidized compound (mCP-tBu), a sulfonylated derivative is obtained as a minor side product, and it is possible that this drawback may be more significant for other organic semiconductors. **1d**_**2**_ can be used to dope semiconductor films through either codeposition from chloroalkane solution or sequential doping using acetonitrile solutions, notably affording a conductivity of over 10^–2^ S cm^–1^ in sequentially doped F8BT (the water stability of which, however, is limited by the reactivity of the F8BT polaron).

## Supplementary Material



## Notes

Synthetic details; additional experimental data including TGA, UV–vis-NIR, NMR, mass spectrometry, and ESR data, along with discussion of the ESR experiments; additional tables and figures of DFT and TD-DFT results for different **1**_**2**_ derivatives (PDF)

## Abbreviations

OSC: organic semiconductor
F_4_TCNQ: 2,3,5,6-tetrafluoro-7,7,8,8-tetracyanoquinodimethane
F_6_TCNNQ: 2,2′-(perfluoronaphthalene-2,6-diylidene)­di­(malononitrile)
CN_6_-CP: hexacyano-1,2,3-trimethylene-cyclopropane
spiro-OMeTAD: 2,2″,7,7″-tetrakis­[*N,N*-di­(4-methoxyphenyl)­amino]-9,9′-spirobifluorene
(N-DMBI)_2_: 2,2′-bis­(4-dimethylaminophenyl)-1,1,3′,3′-tetrahydro-2,2′-bibenzo­[*d*]­imidazole
(DMBI)_2_: 1,1,3″,3′-tetrahydro-2,2″-bibenzo­[*d*]­imidazole
DFT: density functional theory
MB: magic blue (tris­(4-bromophenyl)­ammoniumyl hexachloroantimonate)
MG: magic green (tris­(2,4-dibromophenyl)­ammoniumyl hexachloroantimonate)
UV–vis-NIR: ultraviolet/visible/near-infrared
rr-P3HT: regioregular poly­(3-hexylthiophene-2,5-diyl)
F8BT: poly­[(9,9-dioctylfluorene-2,7-diyl)-*alt*-(benzo­[2,1,3]­thiadiazol-4,7-diyl)]
TAPC: 1,1-bis­[4-[*N,N*-di­(p-tolyl)­amino]­phenyl]­cyclohexane
mCP-tBu: 1,3-bis­(3,6-di-*tert*-butyl-9*H*-carbazol-9-yl)­benzene
PE_2_ and EPE-1.5E: see Chart 2
HOMO: highest occupied molecular orbital
LUMO: lowest unoccupied molecular orbital
