# Supplementary material for: A Bis(arenesulfonyl) Peroxide, an Ambient-Stable Oxidant, Is a Strong p‑Dopant for Organic Semiconductors
Source: Chem Mater. 2026 Jul 10;38(14):7104–16. doi: 10.1021/acs.chemmater.6c00231 (PMC13420587; doi:10.1021/acs.chemmater.6c00231)
Supplement: Supplementary file 1 [file cm6c00231_si_001.pdf]

Supporting Information

A Bis(arenesulfonyl) Peroxide, an Ambient-Stable Oxidant, is a Strong p-Dopant for Organic Semiconductors

*Suman Kuila[†], Aniruddha Basu[†], Megan R. Brown[‡], Spencer J. Gilman[§], Junxiang Zhang[†], Kevin Singewald[^], Glenn Millhauser[^], Chad Risko[‡], John R. Reynolds[§], Seth R. Marder[†,¶,#], Stephen Barlow[†,#,]**

[†]RASEI, University of Colorado Boulder, Boulder, Colorado 80309, United States

Department of Chemistry & Center for Applied Energy Research (CAER), University of Kentucky, Lexington, Kentucky, 40506, United States

[§]School of Chemistry and Biochemistry, School of Materials Science and Engineering, Center for Organic Photonics and Electronics, Georgia Tech Polymer Network, Georgia Institute of Technology, Atlanta, Georgia 30332, United States

[^]Department of Chemistry and Biochemistry, University of California, Santa Cruz, California 95064, United States

[¶]Departments of Chemistry and of Chemical and Biological Engineering, University of Colorado Boulder, Boulder, Colorado 80309, United States

CONTENTS

1. Synthetic Details pS3
   1.1. General Preparation for Bis(arenesulfonyl) Peroxides pS3
   1.2. 1.2. Bis(3,5-bis(trifluoromethyl)benzenesulfonyl) Peroxide, $\mathbf{1d_2}$ pS3
   1.3. Bis(4-cyanobenzenesulfonyl) Peroxide, $\mathbf{1e_2}$ pS4
   1.4. Tetrabutylammonium 3,5-Bis(trifluoromethyl)benzenesulfonate, $Bu_4N^+\mathbf{1d}^-$ pS4
   1.5. 1,3-Bis(3,6-di-*tert*-butyl-9*H*-carbazol-9-yl)benzene, mCP-tBu pS7
2. Additional Experimental Data pS9
   2.1. TGA Data pS9
   2.2. Additional UV-vis.-NIR Data pS9
   2.3. NMR and Mass Spectroscopy Data for Solution Reactivity pS13
   2.4. Representative Electrical Data pS18
   2.5. ESR Data pS19
3. Additional Computational Data pS21
4. References for Supporting Information pS28

1. SYNTHETIC DETAILS

1.1. General Preparation for Bis(arenesulfonyl) Peroxides

We generally followed literature procedures and precautions described in the SI of ref. 1, specifically avoiding the use of metal spatulas while manipulating these compounds. Specifically, the appropriate arenesulfonyl chloride (1.0 equiv.) dissolved in $CHCl_3$ (0.35 mL/mmol) was added to a stirred solution of K_2CO_3 (1.25 equiv.) in H_2O/EtOH (2:1, 4.2 mL/mmol)/30% aqueous H_2O_2 (0.4 mL/mmol) at –20 °C. The solution was stirred at 1500 rpm for 2 min (**Note**: in our experience it is essential that the reaction mixture be stirred vigorously to ensure mixing between the aqueous and organic layers), then EtOH (2 mL/mmol) was added using the same stirring conditions (**Note**: again, we find that the reaction mixture must be stirred vigorously to ensure mixing between the aqueous and organic layers). After a further 15 min stirring the suspension was diluted with H_2O (5 mL/mmol) and filtered. The solid was washed with H_2O and dried by suction filtration on a sintered glass funnel. Finally, the resulting white solid was dried under high vacuum at room temperature for 48 h. **Note:** ref. 1 warns not to "excessively dry" samples due to risk of explosion, but does not qualify what is meant by "excessively"; however, we did not encounter any signs of instability when drying under the conditions we specify here. Data for the two dimers are given below and in Figures S1-5; ^{1}H and ^{19}F NMR data are consistent with the previous report,[1] while ^{13}C NMR data have not previously been reported for these compounds.

1.2. Bis(3,5-bis(trifluoromethyl)benzenesulfonyl) Peroxide, 1d$_2$

White solid. Yield = 7.4 g (79%) from 10 g of the corresponding $ArSO_2Cl$ derivative. ^{1}H NMR (400 MHz, $CDCl_3$): δ 8.40 (m, 4H), 8.29 (m, 2H). ^{19}F{^{1}H} NMR (282 MHz, $CDCl_3$): δ –63.01. ^{13}C{^{1}H} NMR (101 MHz, $CDCl_3$): δ 135.43, 134.64 (q, J = 36 Hz), 129.87 (q, J = 3 Hz), 129.72 (sept, J = 4 Hz), 126.16 (q, J = 276 Hz).

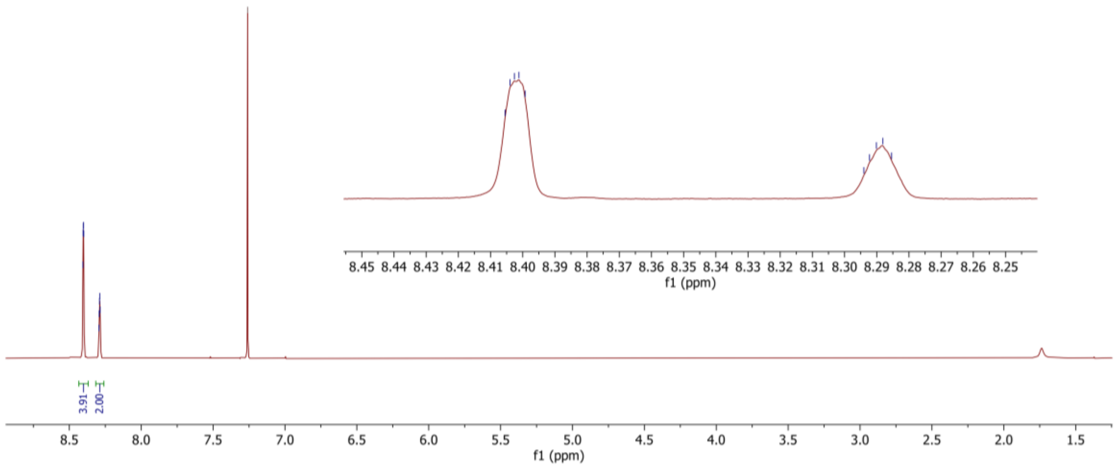

Fig. S1. ^{1}H NMR spectrum of **1d$_2$** in $CDCl_3$.

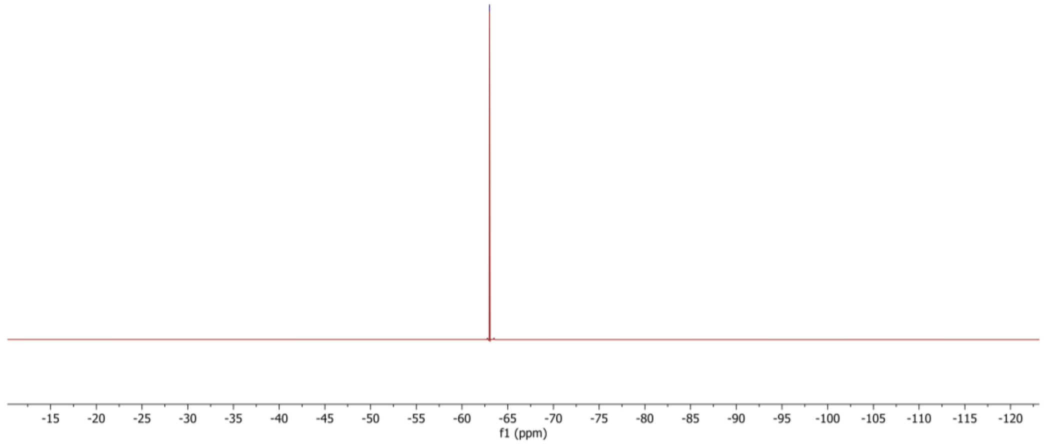

Fig. S2. ^{19}F{^{1}H} NMR spectrum of **1d$_2$** in $CDCl_3$.

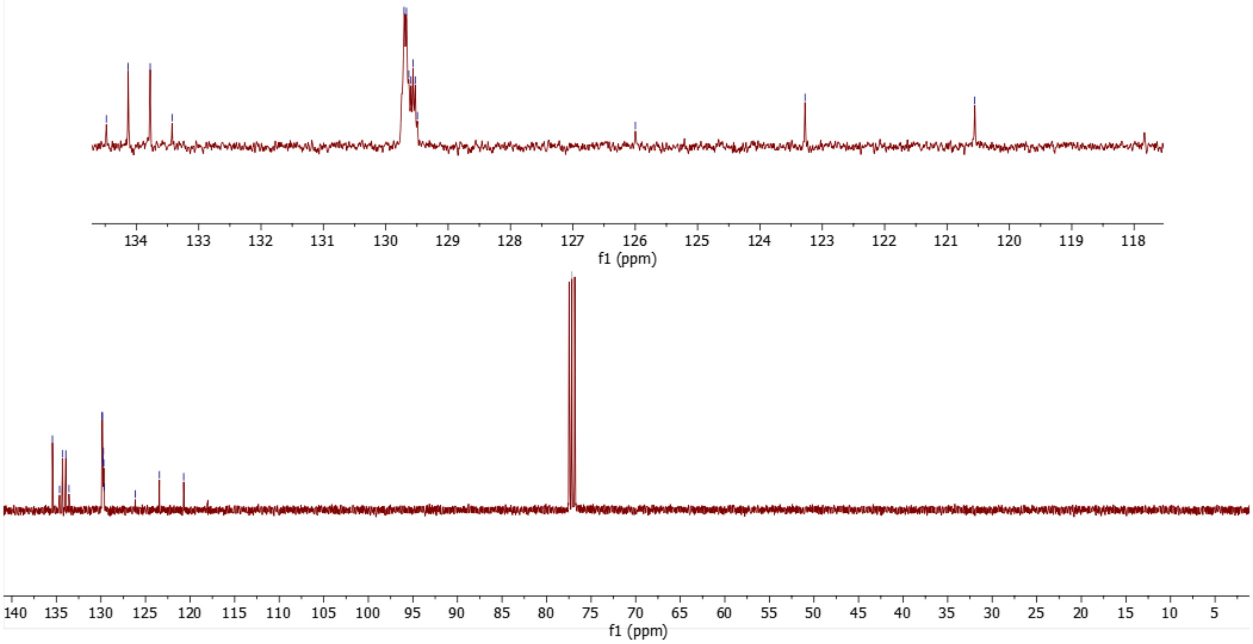

Fig. S3. ^{13}C{^{1}H} NMR spectrum of **1d$_2$** in $CDCl_3$.

1.3. Bis(4-cyanobenzenesulfonyl) Peroxide, 1e$_2$.

White solid. Yield =156 mg (21%) from 400 mg of the corresponding $ArSO_2Cl$ derivative. ^{1}H NMR (400 MHz, $CDCl_3$): δ 8.16-8.07 (m, 4H), 8.01-7.92 (m, 4H). ^{13}C{^{1}H} NMR (101 MHz, $CDCl_3$ plus trace of CD_3CN to increase solubility): δ 136.19, 133.33, 129.97, 119.35, 116.52.

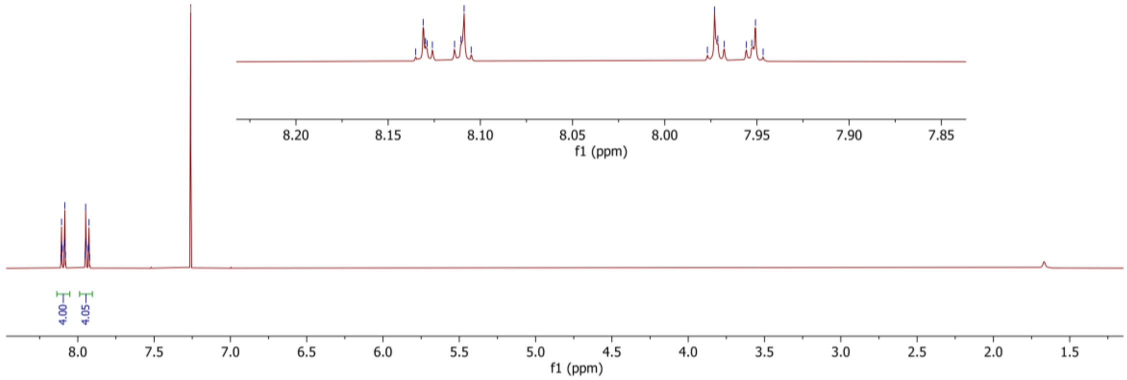

Fig. S4. ^{1}H NMR spectrum of **1e$_2$** in $CDCl_3$.

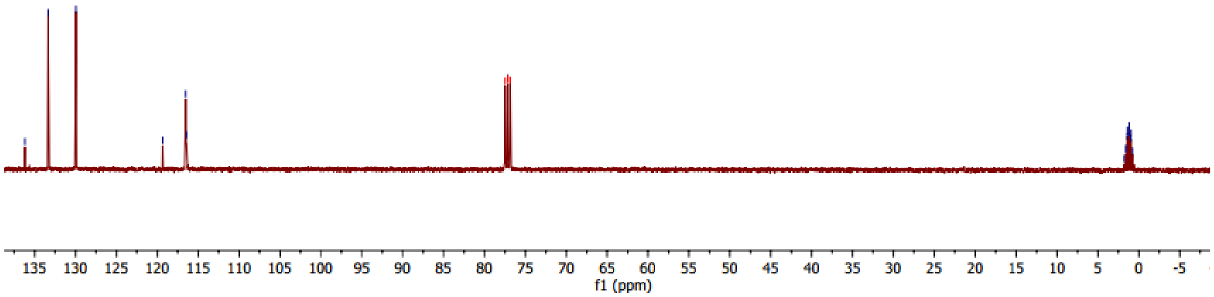

Fig. S5. ^{13}C{^{1}H} NMR spectrum of **1e$_2$** in $CDCl_3$ / CD_3CN.

1.4. Tetrabutylammonium 3,5-Bis(trifluoromethyl)benzenesulfonate, Bu_4N^+1d$^-$.

The synthesis was adapted from a general procedure for converting $ArSO_2Cl$ species to the corresponding $Bu_4N^+ArSO_3^-$ derivatives.[2] Tetrabutylammonium hydroxide (1.1 mL of a 1.0 M MeOH solution, 1.1 mmol) was added slowly to a stirred solution of 3,5-bis(trifluoromethyl)benzenesulfonyl chloride (350 mg, 1.12 mmol) in MeOH (1 mL) under air at room temperature. The solution was stirred for 1 h and evaporated under reduced pressue. The mixture was redissolved in CH_2Cl_2 (10 mL) and washed with H_2O (3 × 10 mL). The organic layer was dried over Na_2SO_4, filtered and concentrated *in vacuo* to afford the title compound as a white solid (321 mg, 54%), NMR data for which are consistent with the literature.[3,4] ^{1}H NMR (400 MHz, $CDCl_3$): δ 8.40 (m, 2H), 7.80 (m, 1H), 3.29 (m, 8H), 1.66 (m, 8H), 1.44 (sext., J = 7.4 Hz, 8H), 1.00 (t, J = 7.3 Hz, 12H). ^{1}H NMR (400 MHz, CD_2Cl_2): δ 8.35 (m, 2H), 7.87 (m, 1H), 3.23 (m,

8H), 1.66 (m, 8H), 1.43 (sext., *J* = 7.4 Hz, 8H), 1.02 (t, *J* = 7.4 Hz, 12H). ^{19}F NMR (376 MHz, $CDCl_3$) *δ* –62.80. ^{19}F NMR (376 MHz, $CDCl_3$) *δ* –63.04.

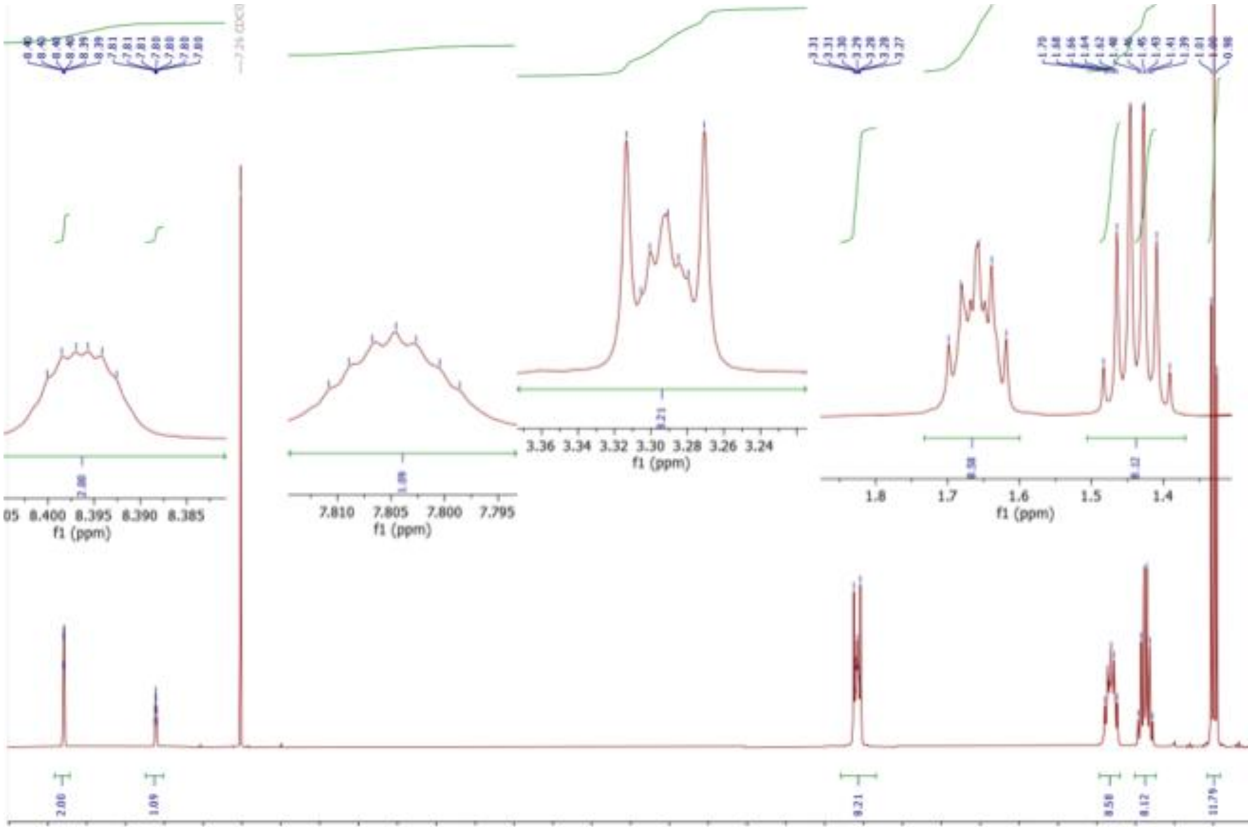

Fig. S6. ^{1}H NMR spectrum of NBu_4^+**1d**$^-$ in $CDCl_3$.

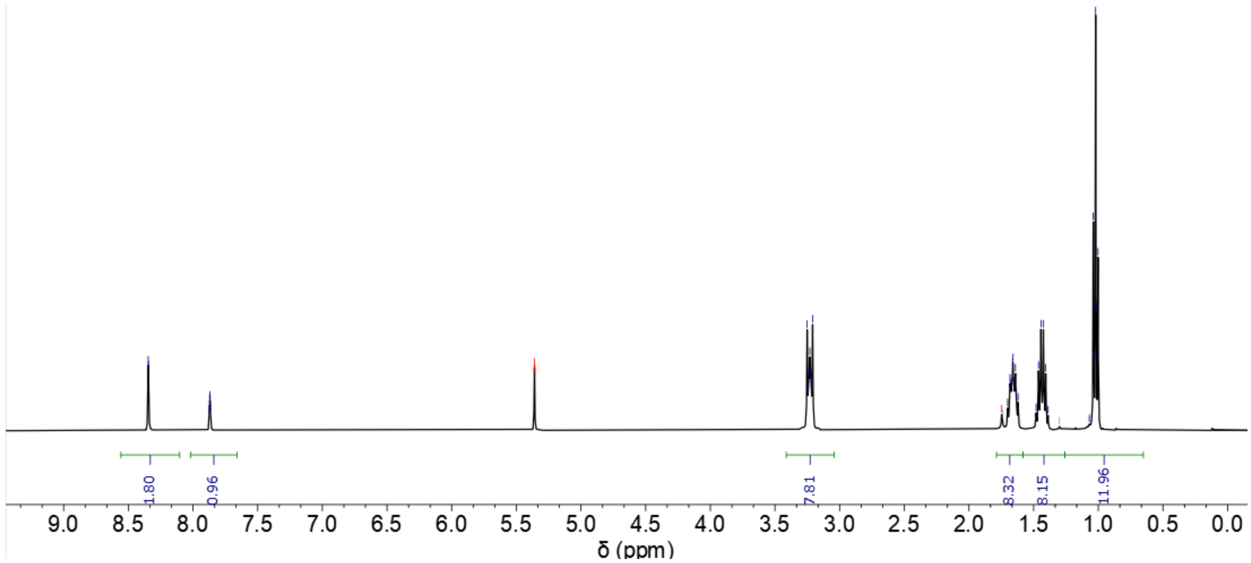

Fig. S7. ^{1}H NMR spectrum of NBu_4^+**1d**$^-$ in CD_2Cl_2.

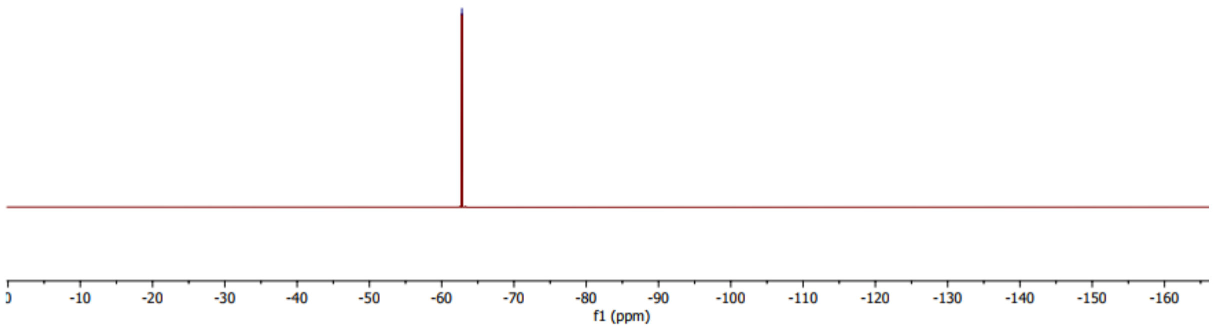

Fig. S8. ^{19}F NMR spectrum of NBu_4^+**1d**$^-$ in $CDCl_3$.

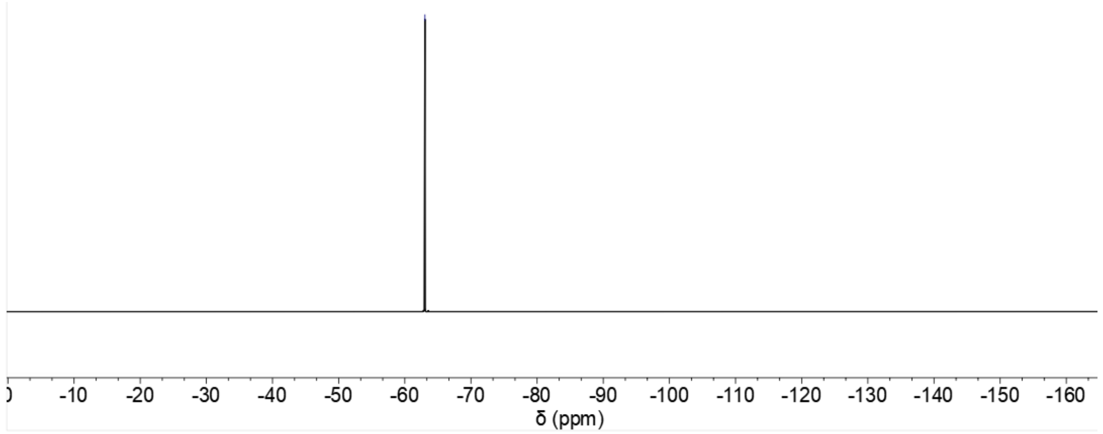

Fig. S9. ^{19}F NMR spectrum of NBu_4^+**1d**$^-$ in CD_2Cl_2.

1.5. 1,3-Bis(3,6-di-*tert*-butyl-9*H*-carbazol-9-yl)benzene, mCP-tBu).

Dry toluene was added to a 100 mL two-necked round-bottom flask charged with 1,3-diodobenzene (1 g, 3 mmol) and 3,6-di-*tert*-butyl-9*H*-carbazole (1.786 g, 6.38 mmol) and the solution was then spurged for 15 min with nitrogen. To this solution, $Pd_2(dba)_3$ (280 mg, 0.3 mmol) and di-*tert*-butyl(methyl)phosphonium tetrafluoroborate (204 mg, 0.6 mmol) were added; the solution was further sparged for 15 min and then ^tBuONa (1.4 g, 15 mmol). The mixture was heated to reflux for 24 h and the progress was monitored by thin-layer chromatography. Upon completion, the resulting mixture was poured into water (20 mL) and extracted with ethyl acetate (3 × 40 mL). The combined organic extracts were dried over anhydrous Na_2SO_4, filtered, and then concentrated under reduced pressure. The residue was purified by column chromatography. (silica gel, dichloromethane/hexane, 3:97 to 1:4, v/v), yielding mCP-tBu as a white solid (1.4 g, 73%). NMR data (following and shown in Fig. S6 and 7) are consistent with data previously reported for the same compound synthesized in a Cu-mediated reaction.[5] ^1H NMR

(400 MHz, $CDCl_3$): δ 8.16 (dd, J = 1.7, 0.9 Hz, 4H), 7.83–7.77 (m, 2H), 7.68–7.62 (m, 2H), 7.48 (m, 8H), 1.48 (s, 36H). $^{13}C\{^1H\}$ NMR (101 MHz, $CDCl_3$): δ 143.21, 139.70, 138.94, 130.95, 124.89, 124.42, 123.79, 123.57, 116.37, 109.20, 34.78, 32.02.

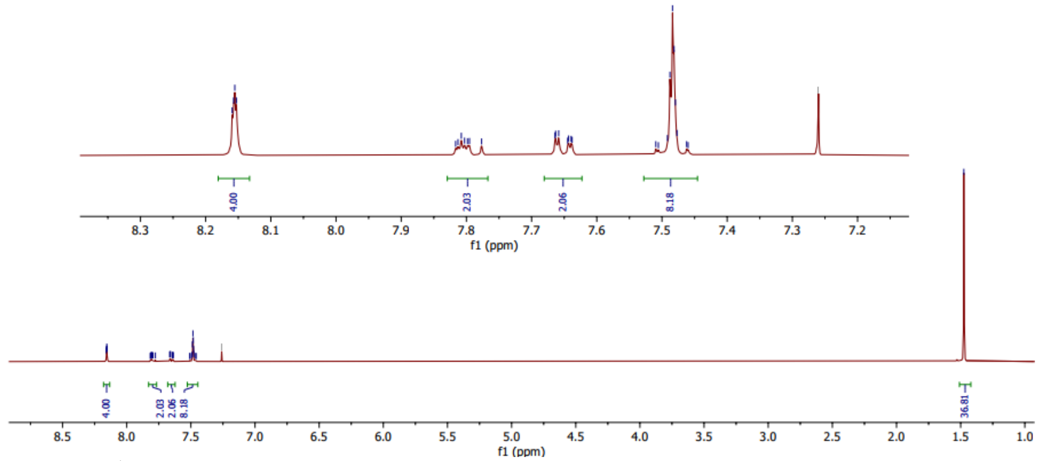

Fig. S10. ^1H NMR spectrum of mCP-tBu in $CDCl_3$.

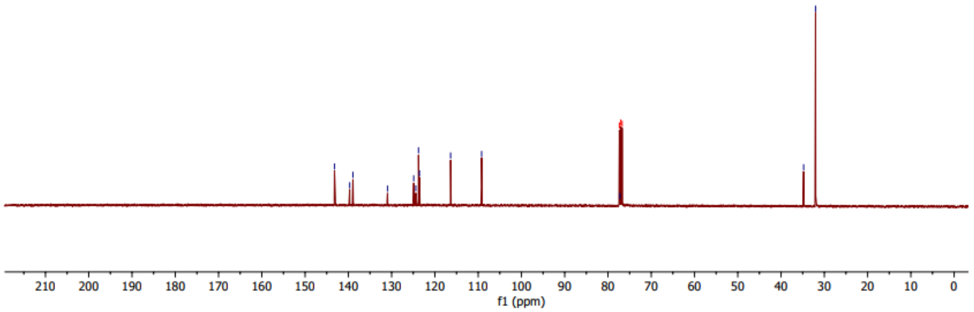

Fig. S11. $^{13}C\{^1H\}$ NMR spectrum of mCP-tBu in $CDCl_3$.

2. ADDITIONAL EXPERIMENTAL DATA

2.1. TGA Data.

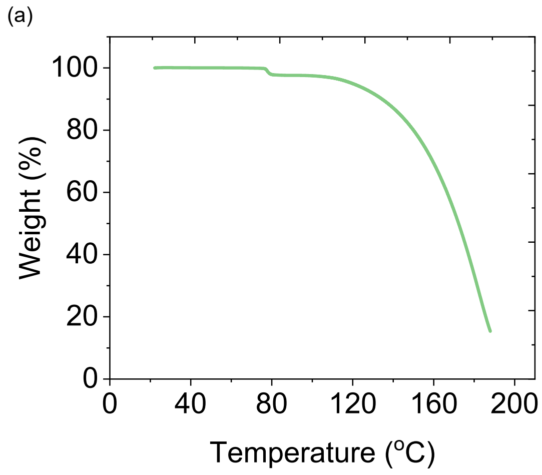

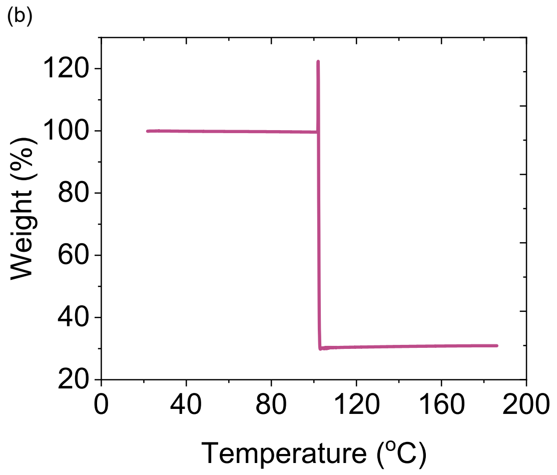

Fig. S12. TGA data for (a) **1d$_2$** and (b) **1e$_2$**, recorded at 20 °C min^{-1}.

2.2. Additional UV-vis.-NIR Data.

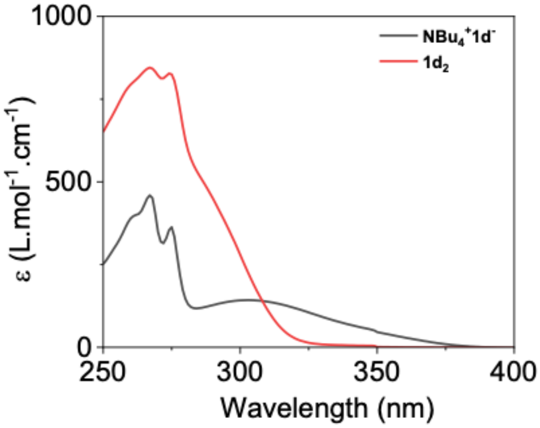

Fig. S13. UV-vis. spectra of **1d$_2$** and NBu$_4^+$**1d$^-$** in MeCN (recorded at concentrations of 1 mM and 2 mM respectively) showing that the spectra of the two species are clearly distinguishable (and thus that the data shown in Fig. 1 indicate the water stability of the dimer). The difference between the two species are also partly consistent with TD-DFT calculations, *e.g.*, regarding of the relative strengths of the long-wavelength features (see Fig. S33).

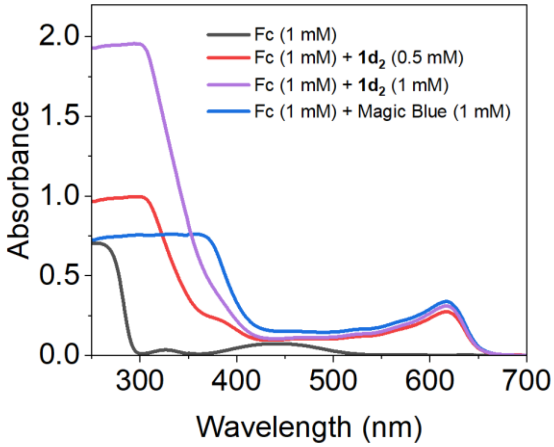

Fig. S14. Optical absorption spectra of ferrocene with and without various oxidants in CH_2Cl_2. The peak at 620 nm is attributed to the ferrocenium ion.[6] The similar absorbances obtained with 0.5 eq. **1d$_2$**, 1 eq **1d$_2$**, and Magic Blue are consistent with **1d$_2$** acting as a net two-electron oxidant.

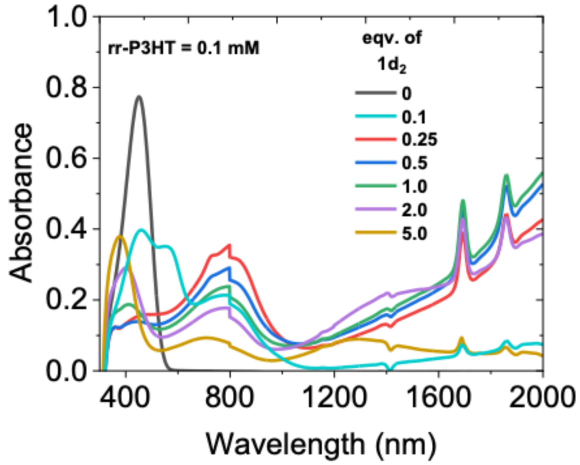

Fig. S15. Optical absorption spectra of 0.1 mM rr-P3HT dissolved in dry dichloromethane and their changes upon doping with increasing amount of **1d$_2$** under inert atmosphere. The features at ca. 800 nm and >2000 nm are attributable to the P3HT polaron, while that seen at ca. 1300-1400 nm for the higher doping levels is attributable to the singlet bipolaron of the polaron.[7]

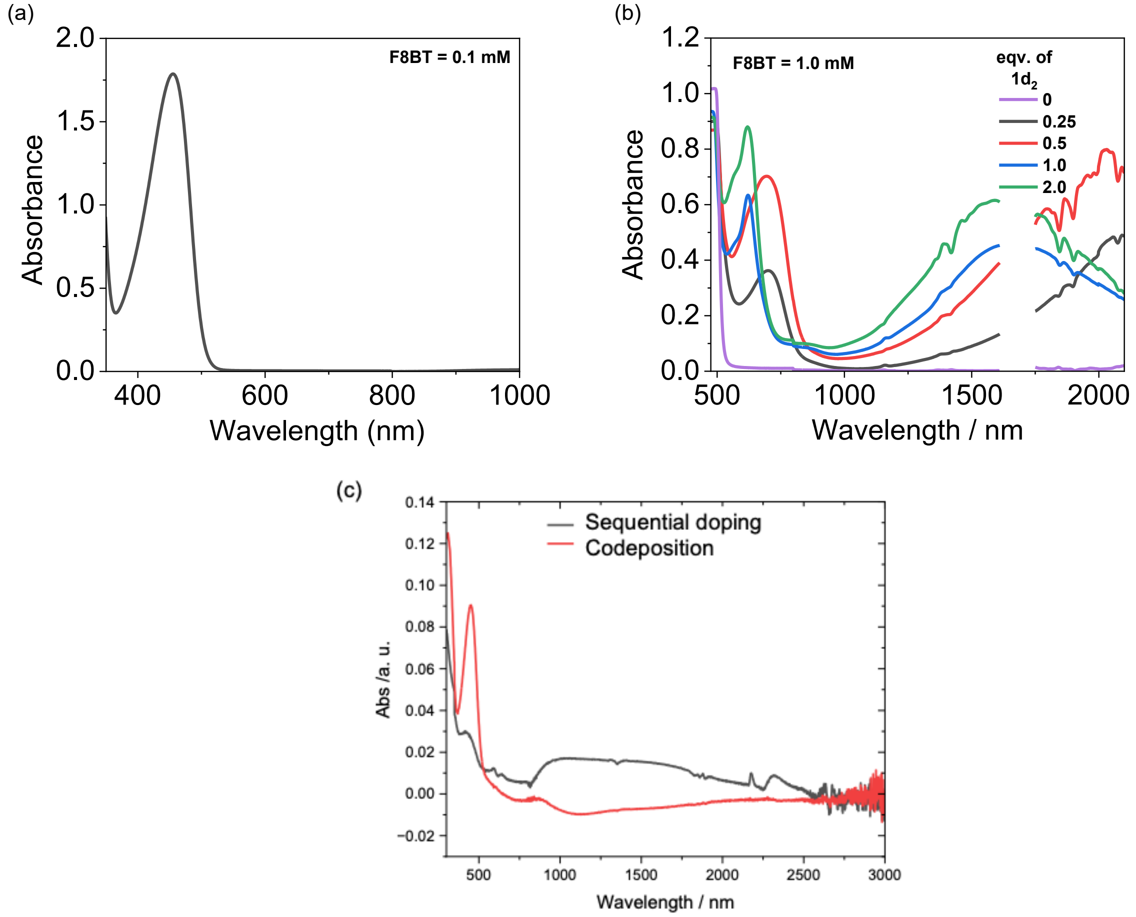

Fig. 16. Optical absorption spectra of (a) 0.1 mM F8BT (no doping), (b) 1 mM F8BT dissolved in dry dichloromethane with increasing amounts of $\mathbf{1c}_2$ under inert atmosphere (a portion of the spectrum, at ca. 1700 nm, being masked off due to the presence of a strong vibronic feature), and (c) films of F8BT doped in the same way as the films for which σ values are reported in Table 1 and cast onto glass. The blue shifts seen with higher doping levels in (b) might reflect the impact of side reactions. In (c) the two polaron bands are clearly seen for the codeposited film. In the sequentially doped film these bands are indistinct, perhaps reflecting the impact of scattering, but the neutral band is clearly more effectively bleached than in the case of the codeposited film.

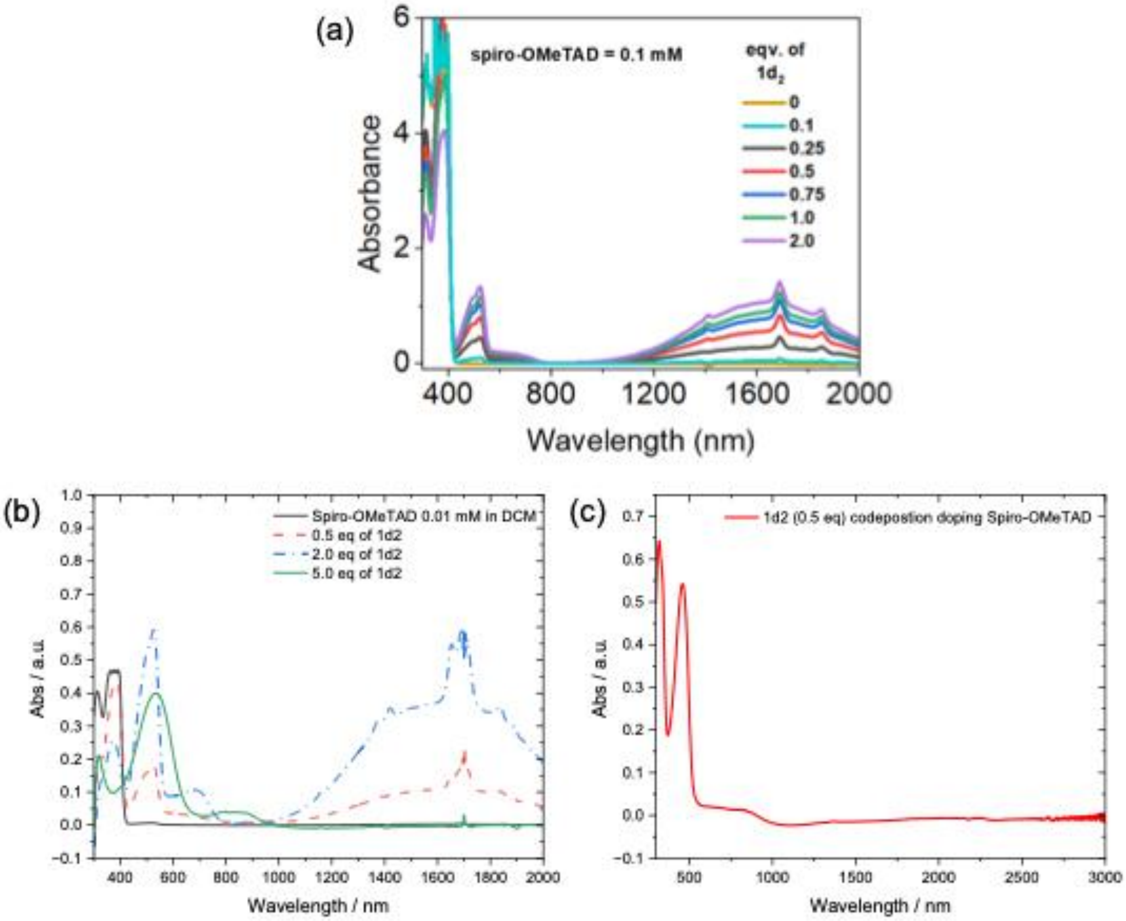

Fig. S17. Optical absorption spectra of spiro-OMeTAD (a) and (b) in dry dichloromethane under different concentration regimes upon doping with increasing amounts of $\mathbf{1d_2}$ under inert atmosphere, and (c) in a doped film on glass prepared analogously to the films for which a σ value is reported in Table 1. The spectra with higher doping ratios in (b) indicate the presence of triply or quadruply charged cations of spiro-OMeTAD.[8]

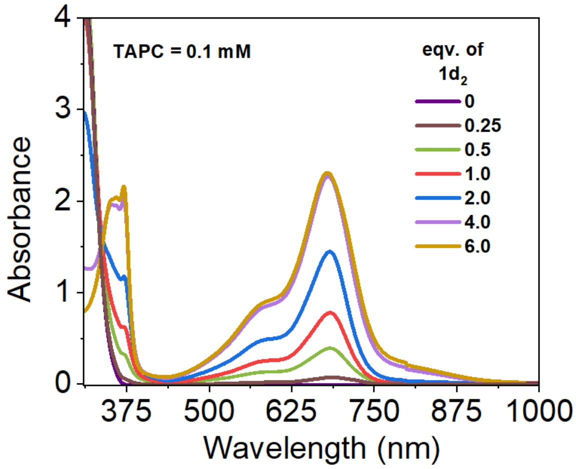

Fig. S18. Optical absorption spectra of TAPC dissolved in dry dichloromethane and their changes upon doping with increasing amount of $\mathbf{1d_2}$ under inert atmosphere.

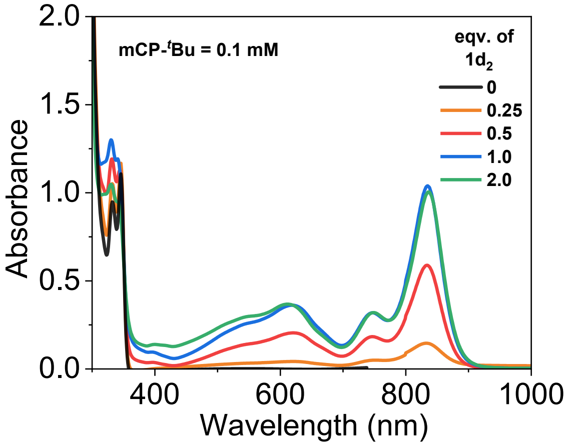

Fig. S19. Optical absorption spectra of 0.1 mM mCP-^{t}Bu dissolved in dry dichloromethane and their changes upon doping with increasing amount of **1d$_2$** under inert atmosphere.

2.3. NMR and Mass Spectroscopy Data for Solution Reactions

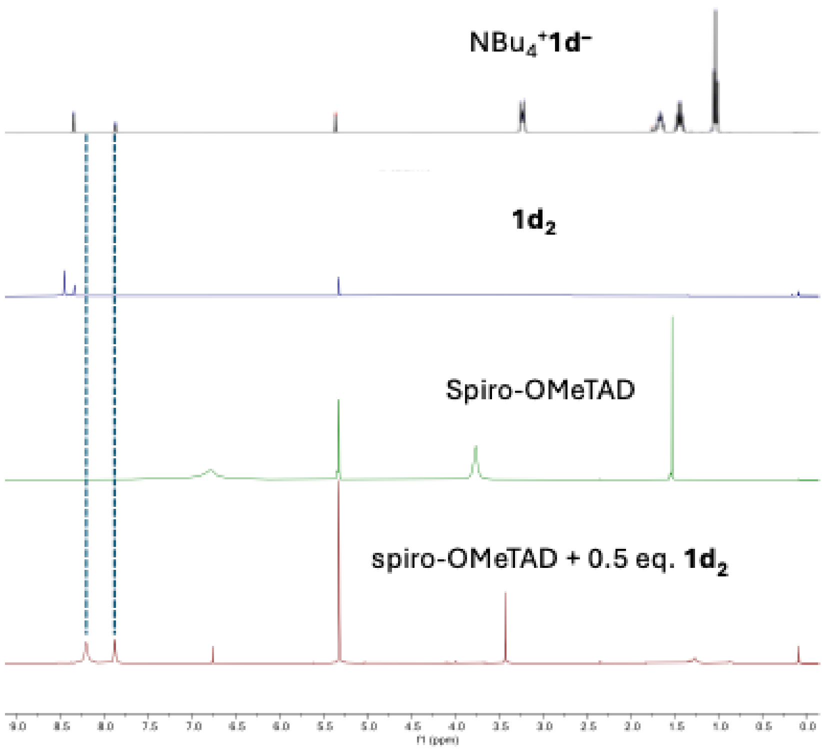

Fig. S20. Comparison of ^{1}H NMR spectra of NBu$_4^+$**1d$^-$**, **1d$_2$**, spiro-OMeTAD, and the reaction product of spiro-OMeTAD + 0.5 eq. **1d$_2$** in CD_2Cl_2. The disappearance of spiro-OMeTAD signals in the spectrum of the reaction mixture is consistent with the formation of spiro-OMeTAD$^{\bullet+}$ indicated by the UV-vis.-NIR spectra shown in Fig. 2 and Fig. S16 (although note a couple of unidentified sharp impurity features are present). Note the signals of the undoped spiro-OMeTAD are broad, likely due to the presence of a small quantity of traces of spiro-OMeTAD$^{\bullet+}$ in rapid electron exchange with the neutral species. The spectrum of the mixture also suggests the only diamagnetic species present, besides the residual non-deuterated solvent, is **1d$^-$**; slight differences in chemical shift from the **1d$^-$** signals of the NBu$_4^+$ salt might be attributable to differences in ion pairing and/or the impact of the paramagnetic spiro-OMeTAD$^{\bullet+}$.

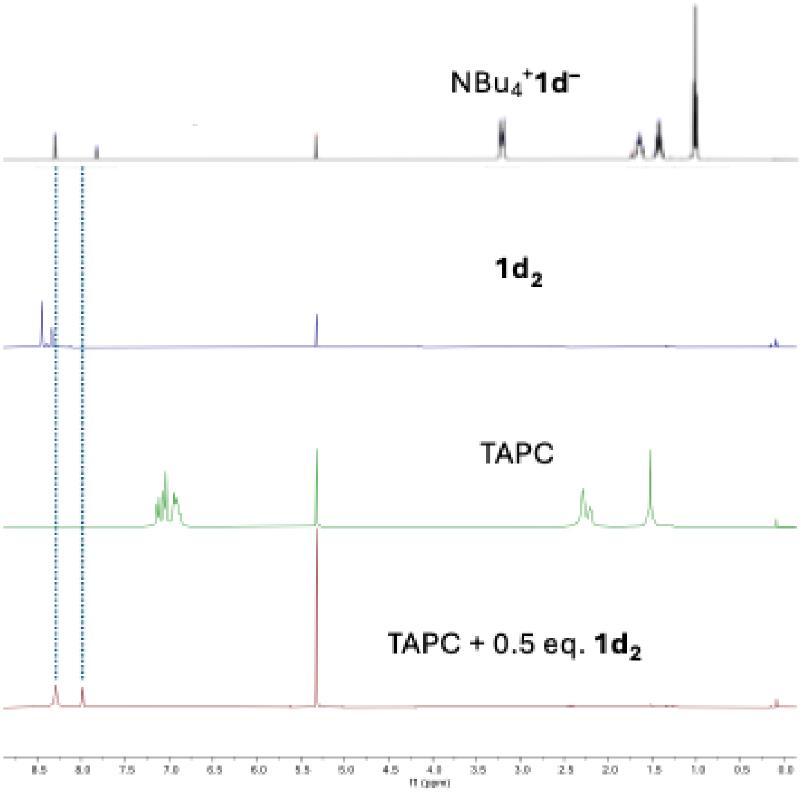

Fig. S21. Comparison of ^{1}H NMR spectra of NBu_4^+**1d**$^-$, **1d$_2$**, TAPC, and the reaction product of TAPC + 0.5 eq. **1d$_2$** in CD_2Cl_2. The disappearance of TPAC signals in the spectrum of the reaction mixture is consistent with the formation of TAPC$^{\bullet+}$ indicated by the UV-vis.-NIR spectra shown in Fig. 2 and Fig. S17. The spectrum of the mixture also suggests the only diamagnetic species present, besides the residual non-protonated solvent, is **1d**$^-$; slight differences in chemical shift from the **1d**$^-$ signals of the NBu_4^+ salt might be attributable to differences in ion pairing and/or the impact of the paramagnetic TAPC$^{\bullet+}$.

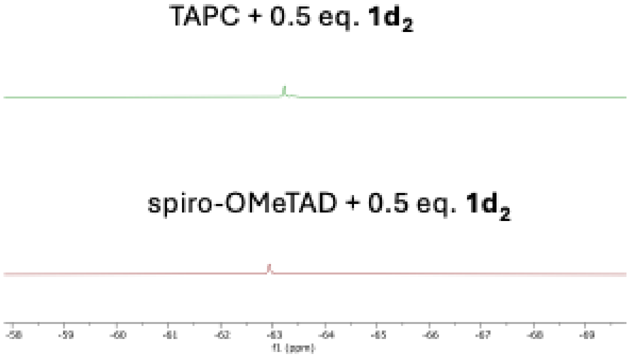

Fig. S22. Comparison of ^{19}F NMR spectra of the reaction products of TAPC and spiro-OMeTAD with 0.5 eq. **1d$_2$** in CD_2Cl_2. The ^{19}F spectra indicate the presence of a single ^{19}F species, but are not very useful at helping distinguish **1d$_2$** from **1d**$^-$ (see Fig. S2 and Fig. S9).

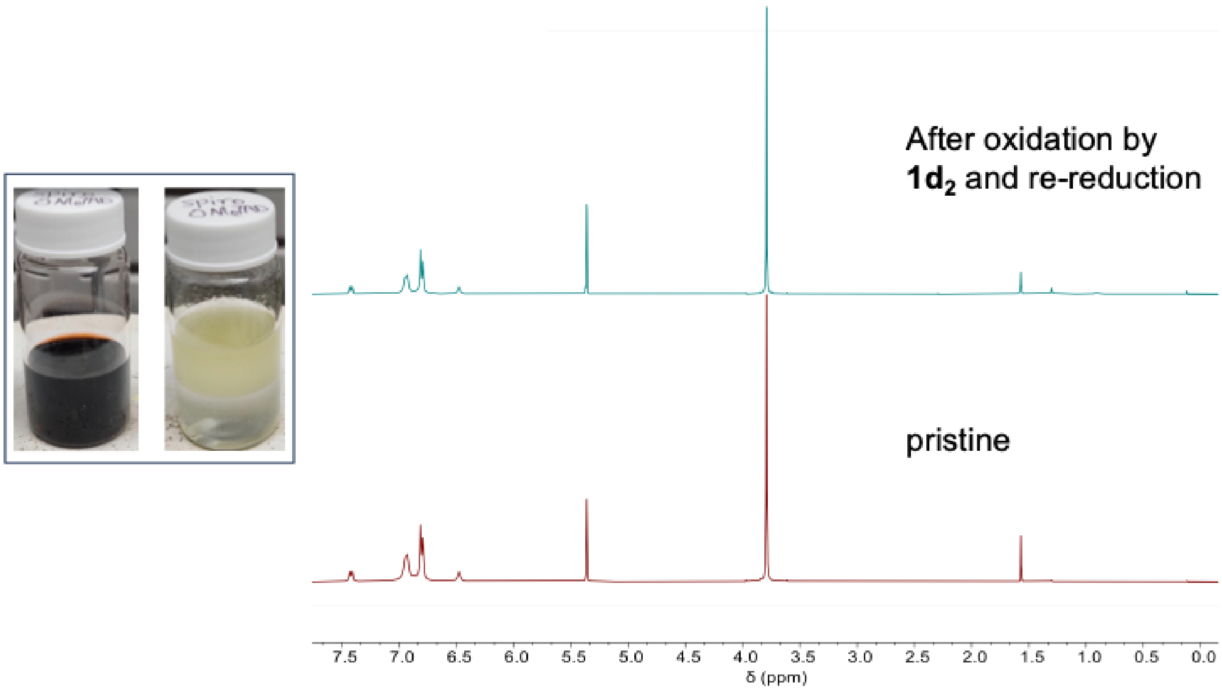

Fig. S23. Comparison of ^{1}H NMR spectra in CD_2Cl_2 of as-received spiro-OMeTAD and a sample oxidized by **$1d_2$** and rereduced by aq. $Na_2S_2O_4$, the similarity indicating spiro-OMeTAD is largely unchanged by oxidation and rereduction. The photographs show spiro-OMeTAD oxidized by **$1d_2$** (left) and after the addition of aq. $Na_2S_2O_4$ (right).

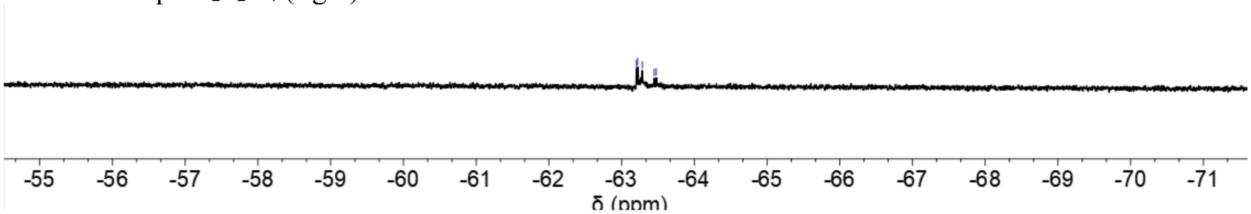

Fig. S24. ^{19}F NMR spectrum of the CD_2Cl_2-soluble extract from spiro-OMeTAD oxidized by **$1d_2$** and rereduced by aq. $Na_2S_2O_4$, indicating the presence of only a very small quantity of F-containing material.

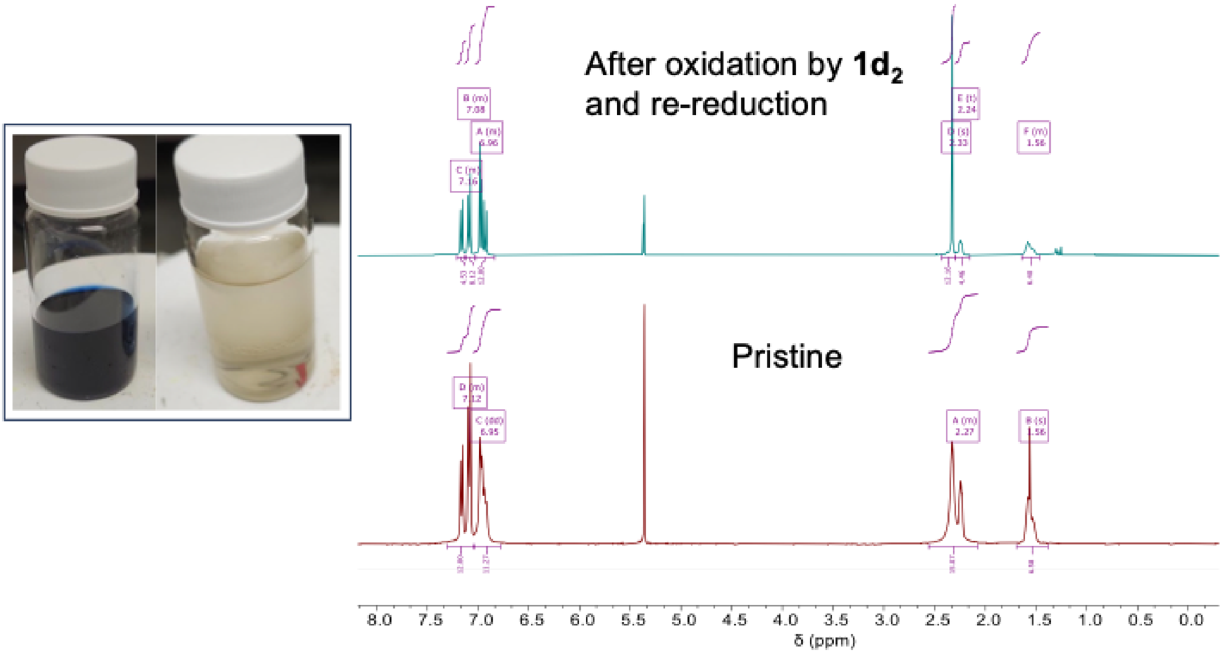

Fig. S25. Comparison of ^{1}H NMR spectra in CD_2Cl_2 of as-received TAPC and a sample oxidized by $\mathbf{1d_2}$ and rereduced by aq. $Na_2S_2O_4$, the similarity indicating TAPC is largely unchanged by oxidation and rereduction (minor differences are attributable to solvents and water). The photographs show TAPC oxidized by $\mathbf{1d_2}$ (left) and after the addition of aq. $Na_2S_2O_4$ (right).

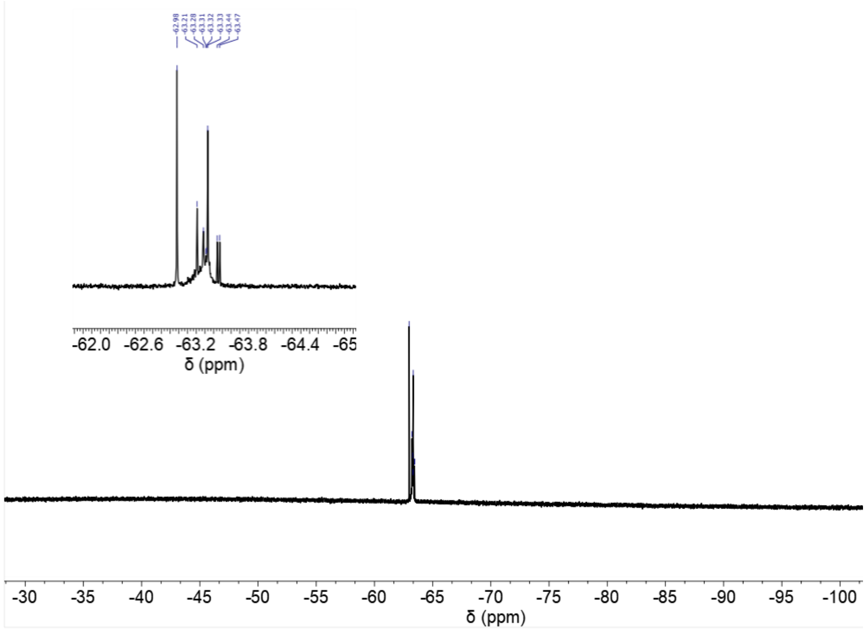

Fig. S26. ^{19}F NMR spectrum of the CD_2Cl_2-soluble extract from TAPC oxidized by $\mathbf{1d_2}$ and rereduced by aq. $Na_2S_2O_4$; note that these spectra are weak and no corresponding aryl signals are seen in the ^{1}H NMR.

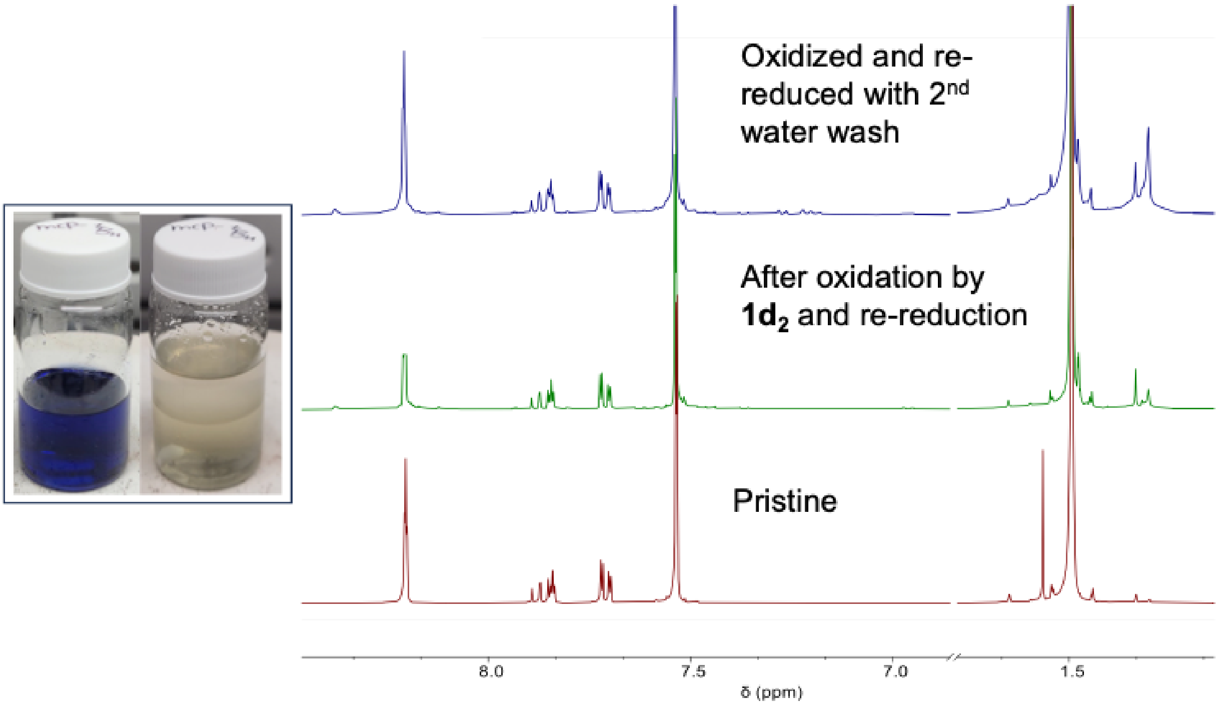

Fig. S27. Comparison of ^{1}H NMR spectra in CD_2Cl_2 of as-synthesized mCP-tBu and a sample oxidized by $\mathbf{1d_2}$ and rereduced by aq. $Na_2S_2O_4$, the similarity indicating mCP-tBu is *largely* unchanged by oxidation and rereduction, but that new diamagnetic species are present after oxidation / rereduction and are not removed by additional washing with water. In particular note new peaks at ca. 1.47 ppm (next to the strong ^{t}Bu resonance) and at ca. 8.38 ppm, along with additional weak features in the range 7.0-7.5 ppm. The photographs show mCP-tBu oxidized by $\mathbf{1d_2}$ (left) and after the addition of aq. $Na_2S_2O_4$ (right).

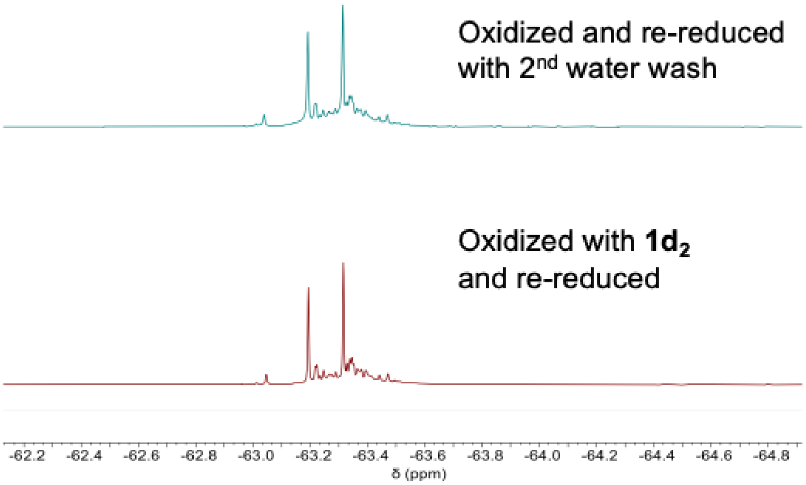

Fig. S28. ^{19}F NMR spectrum of the CD_2Cl_2-soluble extract from mCP-tBu oxidized by $\mathbf{1d_2}$ and rereduced by aq. $Na_2S_2O_4$, showing multiple signals, two of which are relatively strong.

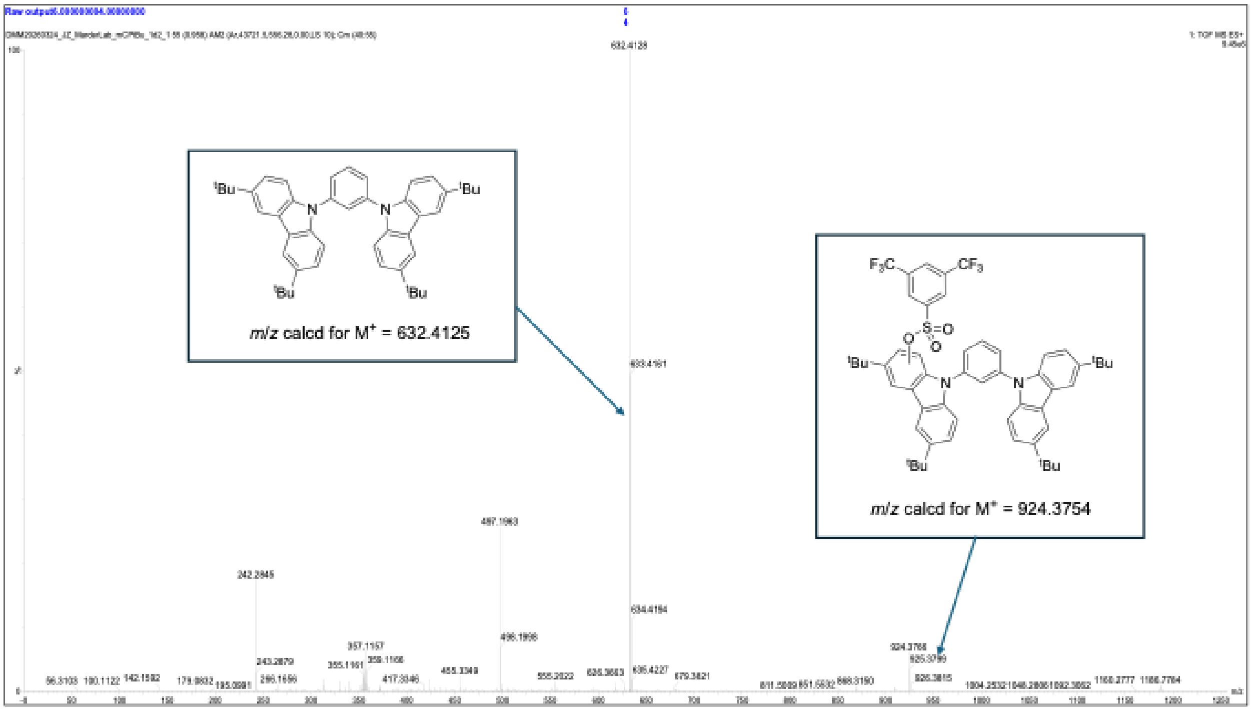

Fig. S29. High-resolution ESI mass spectrum acquired on a Waters instrument of the CH_2Cl_2 extract obtained after doping of mCP-tBu with **1d₂** and rereduction, with assignments of the main peaks (at 632.4128 and 924.3766) along with calculated exact masses for the corresponding positively charged ions. The $ArSO_3$-substituted derivative accounts for ca. 3-5% relative to unreacted mCP-tBu assuming a similar ionization efficiency.

2.4. Representative Electrical Data.

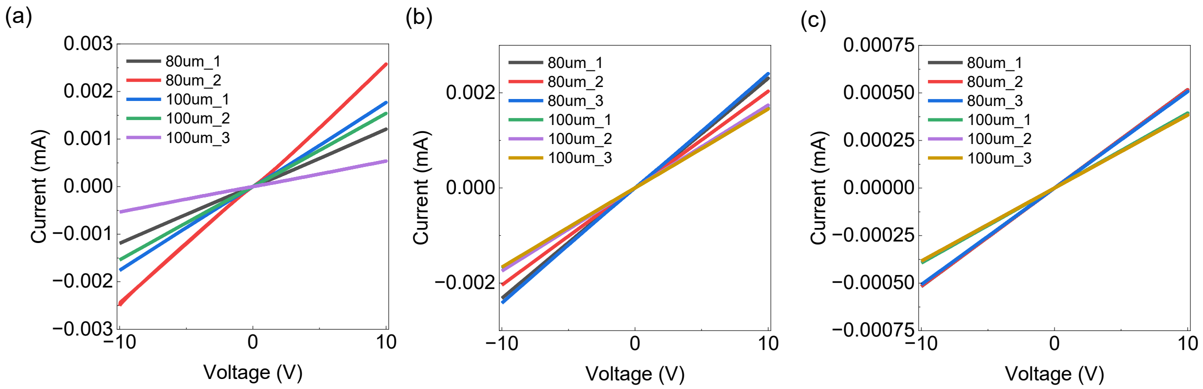

Fig. S30. Current-voltage characteristics for pristine and 50%-doped films of (a) rr-P3HT, (b) spiro-OMeTAD and (c) F8BT, with **1d$_2$** on interdigitated Cr/Au electrodes.

2.5. ESR Data.

No ESR signal could be detected for a 5 mM solution of **1d**$_2$ in MeCN, either at room temperature or at 80 °C. We estimated the limit of detection was ca. 100 nM and thus assume [**1d**$^•$] < 10^{-7} M, [**1**$_2$] ~ 5×10^{-3} M, and, therefore,

$$K_{\mathrm{diss}} = [\mathbf{1d}^•]^2 / [\mathbf{1d}_2] < 2 \times 10^{-12}\ \mathrm{M}$$

and thus, at 298 K,

$$\Delta G_{\mathrm{diss}} = -RT\ln(K_{\mathrm{diss}}) > +15.9\ \mathrm{kcal\ mol^{-1}}$$

and, at 353 K,

$$\Delta G_{\mathrm{diss}} > +18.9\ \mathrm{kcal\ mol^{-1}}$$

which is roughly consistent with the DFT estimate of $\Delta G_{\mathrm{diss(298K)}}$ = +17.9 kcal mol^{-1} (see Table S1). As noted in the experimental section (SI section 1.2.4), we also carried out measurements in which **1d**$_2$ was mixed with DMPO as a spin trap (Fig. S16). In this case a radical signal was seen, the appearance of which is similar to that of other [DMPO-X]$^•$ adducts such as those with X = OH or OOH,[9] and might be assignable to the X = OSO_2Ar (Ar = 3,5-$(CF_3)_2C_6H_3$) adduct. While such a product might be formed by trapping of equilibrium concentrations of **1c**$^•$ by DMPO, it might also be formed by a redox reaction, whereby DMPO is oxidized to DMPO$^{•+}$ and subsequently reacts with **1c**$^-$ or water (and in the proposed mechanism for photoinduced formation of [DMPO-OH]$^•$ in water). Furthermore, "DMPO dimer" can also be formed by proton loss from DMPO$^{•+}$, followed by trapping by DMPO[10] and shows a similar ESR spectrum to that detected in this work.[11] However, the intensity of the radical signal in the present work showed a non-straightforward dependence on temperature and time (see Fig. S30); further elucidation of the reaction is beyond the scope of this work.

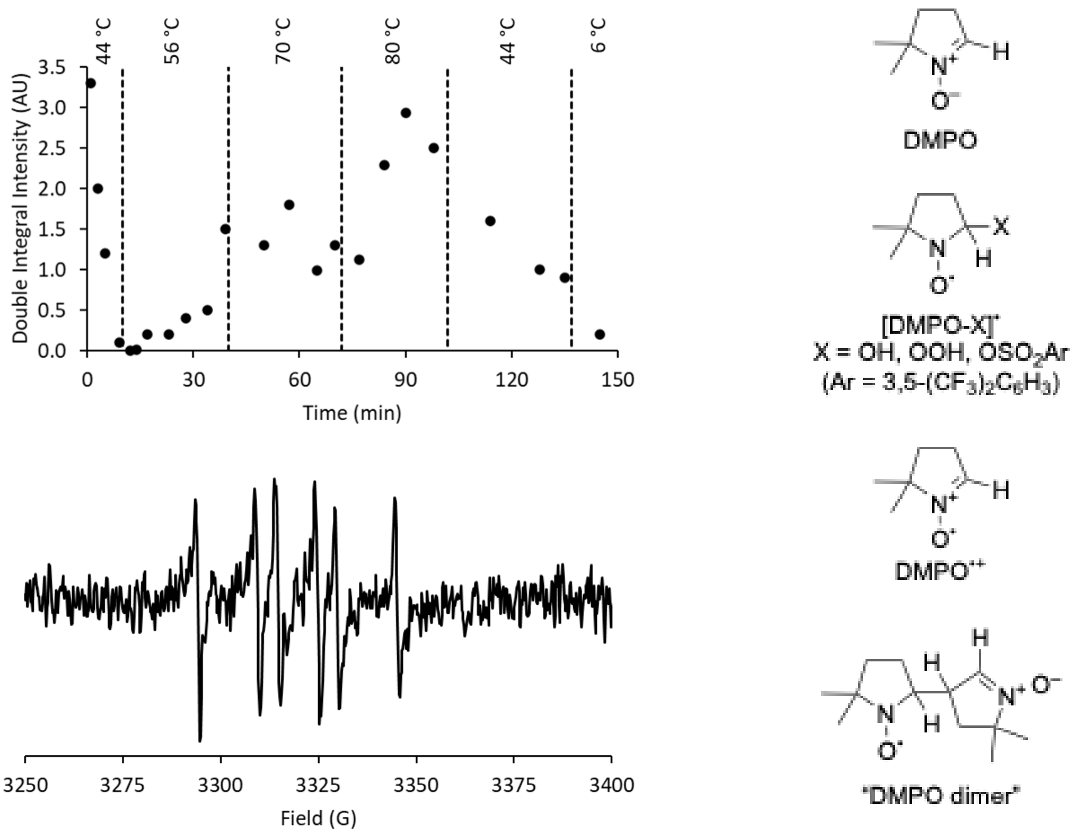

Fig. S31. Left top: evolution of the ESR intensity for a sample of **1d₂** (5 mM) in the presence of a spin-trap, DMPO (100 mM) measured at acetonitrile:water (9:1). Left: the ESR spectrum obtained at 80 °C. Right: chemical structure of DMPO, some known and possible products of radical trapping, and known oxidation products of DMPO.

3. ADDITIONAL COMPUTATIONAL DATA

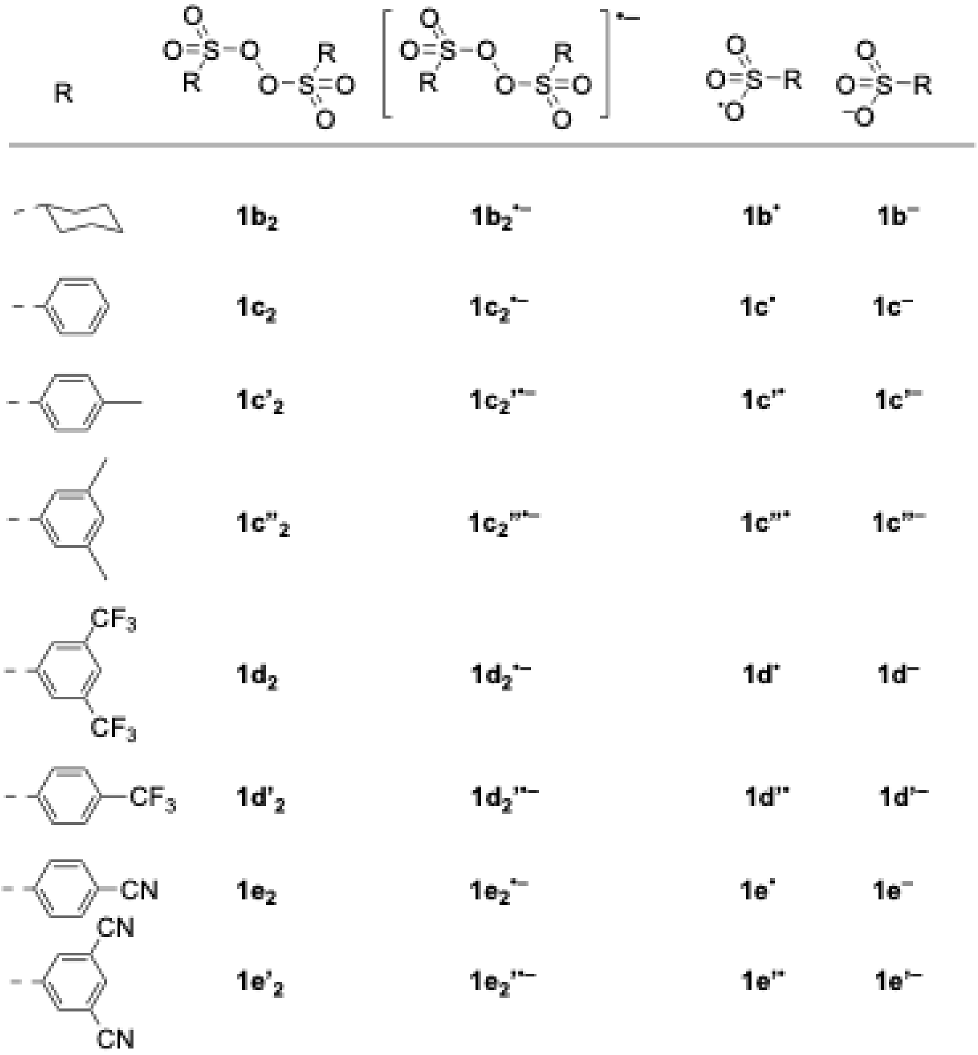

Fig. S32. Numbering scheme for compounds referred to in section 3 of the Supporting Information.

Table S1. M06-2X/6-311+G(3df,2p) Reaction Energies (ΔE, kcal mol^{-1}) and Reaction Gibbs Free Energies (ΔG, kcal mol^{-1}) for Reactions of Bis(Sulfonyl) Peroxide Derivatives in MeCN.

	Reaction	Derivative, R							
		**b**	**c**	**c'**	**c''**	**d**	**d'**	**e**	**e'**
		C_6H_{11}	Ph	4-MeC_6H_4	3,5-$Me_2C_6H_3$	3,5-$(CF_3)_2C_6H_3$	4-$(CF_3)C_6H_3$	4-(NC)C_6H_4	3,5-$(NC)_2C_6H_3$
ΔE	$\mathbf{1}_2 \rightarrow 2\mathbf{1}^{\bullet}$	78.5	34.6	22.5	34.6	34.3	39.8	39.6	34.0
	$\mathbf{1}_2 + e^- \rightarrow \mathbf{1}_2^{\bullet-}$	–132.7	–131.9	-130.8	–130.8	–138.7	–135.4	–136.7	–140.7
	$\mathbf{1}_2^{\bullet-} \rightarrow \mathbf{1}^{\bullet} + \mathbf{1}^-$	28.4	3.5	-2.7	3.4	5.0	14.9	6.9	4.9
	$\mathbf{1}^{\bullet} + e^- \rightarrow \mathbf{1}^-$	–182.7	–163.0	–155.9	–161.9	–168.0	–160.3	–169.4	–169.9
	$0.5\mathbf{1}_2 + e^- \rightarrow \mathbf{1}^-$	–143.5	–145.7	–133.4	–144.6	–150.9	–140.4	–149.6	–152.9
ΔG	$\mathbf{1}_2 \rightarrow 2\mathbf{1}^{\bullet}$	16.8	18.0	4.3	17.8	17.9	23.1	22.8	17.0
	$\mathbf{1}_2 + e^- \rightarrow \mathbf{1}_2^{\bullet-}$	–134.0	–134.6	–134.2	–132.8	–141.0	–137.2	–139.2	–143.5
	$\mathbf{1}_2^{\bullet-} \rightarrow \mathbf{1}^{\bullet} + \mathbf{1}^-$	–8.2	–9.0	–15.4	–10.4	–8.3	–6.5	–6.0	–7.9
	$\mathbf{1}^{\bullet} + e^- \rightarrow \mathbf{1}^-$	–159.0	–161.6	–153.9	–161.0	–167.1	–166.8	–168.1	–168.4
	$0.5\mathbf{1}_2 + e^- \rightarrow \mathbf{1}^-$	–150.6	–152.6	–151.8	–152.1	–158.2	–155.3	–156.7	–159.9

Table S2. M06-2X/6-311+G(3df,2p) Reaction Energies (ΔE, kcal mol^{-1}) and Reaction Gibbs Free Energies (ΔG, kcal mol^{-1}) for Reactions of Bis(Sulfonyl) Peroxide Derivatives in $CHCl_3$

	Reaction	Derivative, R							
		**a'** C_6H_{11}	**b** Ph	**b'** 4-MeC_6H_4	**b''** 3,5-$Me_2C_6H_3$	**c** 3,5-$(CF_3)_2C_6H_3$	**c'** 4-$(CF_3)C_6H_3$	**d** 4-(NC)C_6H_4	**d'** 3,5-$(NC)_2C_6H_3$
ΔE	$\mathbf{1_2} \rightarrow 2\mathbf{1}^{\bullet}$	33.7	34.8	31.4	34.7	*a*	42.2	42.1	*a*
	$\mathbf{1_2} + e^- \rightarrow \mathbf{1_2}^{\bullet-}$	–122.9	–121.4	–119.9	–118.5	*a*	-126.8	-128.9	*a*
	$\mathbf{1_2}^{\bullet-} \rightarrow \mathbf{1}^{\bullet} + \mathbf{1}^-$	7.7	4.4	2.4	2.7	6.8	*b*	9.9	13.4
	$\mathbf{1}^{\bullet} + e^- \rightarrow \mathbf{1}^-$	–148.9	–151.8	–149.0	–150.5	-159.1	*b*	-161.0	-161.8
	$0.5\mathbf{1_2} + e^- \rightarrow \mathbf{1}^-$	–132.1	–134.4	-133.3	–133.2	*a*	*b*	–140.0	*a*
ΔG	$\mathbf{1_2} \rightarrow 2\mathbf{1}^{\bullet}$	16.7	18.5	14.8	17.8	*a*	27.0	25.6	*a*
	$\mathbf{1_2} + e^- \rightarrow \mathbf{1_2}^{\bullet-}$	–123.4	–121.5	–121.2	–120.0	*a*	–127.8	–130.3	*a*
	$\mathbf{1_2}^{\bullet-} \rightarrow \mathbf{1}^{\bullet} + \mathbf{1}^-$	–7.2	–10.3	–11.5	–10.0	–6.1	*b*	–3.6	–0.9
	$\mathbf{1}^{\bullet} + e^- \rightarrow \mathbf{1}^-$	–147.3	–150.3	–147.5	–147.9	–157.3	*b*	–159.5	–159.6
	$0.5\mathbf{1_2} + e^- \rightarrow \mathbf{1}^-$	–139.0	–141.1	-140.1	–139.0	*a*	*b*	–146.7	*a*

[a] Geometries for relevant $\mathbf{1_2}$ derivatives did not converge. [b] Geometries for relevant $\mathbf{1}^-$ did not converge.

Table S3. M06-2X/6-311+G(3df,2p) Central O—O Bond Lengths (*R*, Å) for Neutral and Reduced Bis(Sulfonyl) Peroxide Derivatives.[a]

Solvent	Quantity	Derivative, R							
		**a'**	**b**	**b'**	**b''**	**c**	**c'**	**d**	**d'**
		C_6H_{11}	Ph	4-MeC_6H_4	3,5-$Me_2C_6H_3$	3,5-$(CF_3)_2C_6H_3$	4-$(CF_3)C_6H_3$	4-(NC)C_6H_4	3,5-$(NC)_2C_6H_3$
MeCN	$R(\mathbf{1_2})$	1.410	1.415	1.415	1.415	1.408	1.412	1.412	1.408
	$R(\mathbf{1_2}^{\bullet-})$	2.106	2.104	2.108	2.107	2.089	2.095	2.093	2.087
	$\Delta R=R(\mathbf{1_2}^{\bullet-})-R(\mathbf{1_2})$	0.696	0.688	0.693	0.691	0.681	0.683	0.681	0.679
$CHCl_3$	$R(\mathbf{1_2})$	1.409	1.412	1.413	1.413	*a*	1.411	1.411	*a*
	$R(\mathbf{1_2}^{\bullet-})$	2.113	2.092	2.091	2.096	2.084	2.090	2.087	2.158
	$\Delta R=R(\mathbf{1_2}^{\bullet-})-R(\mathbf{1_2})$	0.705	0.680	0.678	0.683	*a*	0.679	0.676	*a*

[a] Geometries for relevant $\mathbf{1_2}$ derivatives did not converge in $CHCl_3$ and so are omitted.

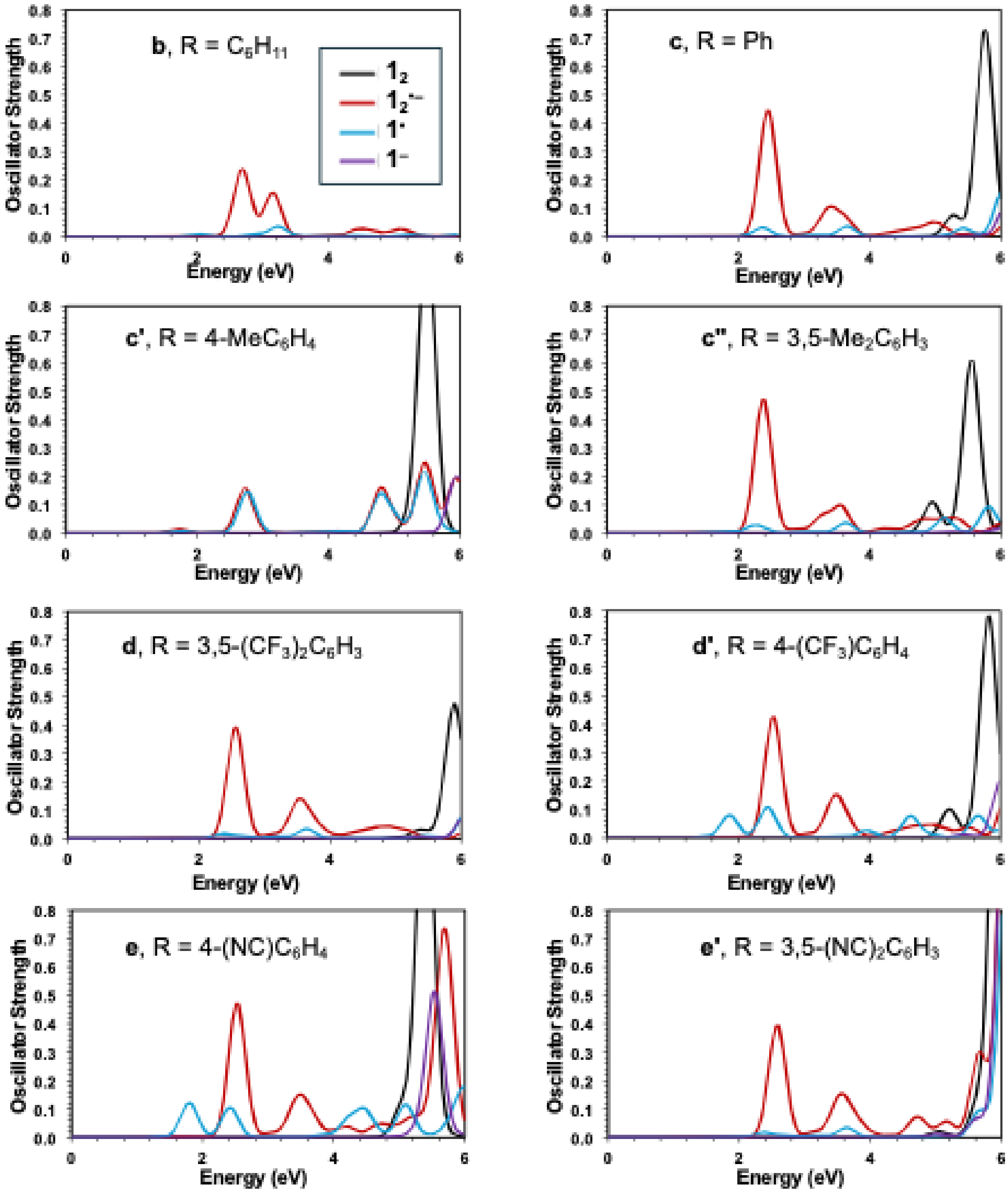

Fig. S33. M06-2X/6-311+G(3df,2p) absorption spectra for bis(sulfonyl) peroxides and related species in MeCN.

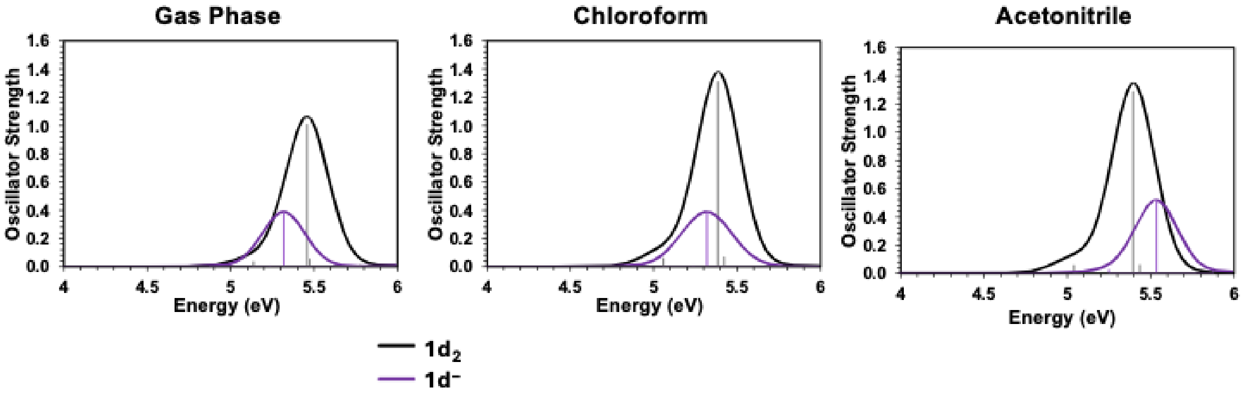

Fig. S34. M06-2X/6-311+G(3df,2p) absorption spectra for **1d$_2$** and **1d$^-$** in the gas phase, $CHCl_3$, and MeCN. Compare with experimental data in Fig. S13, noting that the relative transition energies for the two species are highly solvent dependent.

Table S4. Lowest Energy Transitions for 1d$^-$ in MeCN from M06-2X/6-311+G(3df,2p) Calculations.

Transition	*E* / eV	*f*	NTOs
S0→S1 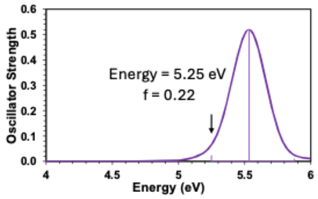	5.25	0.0222	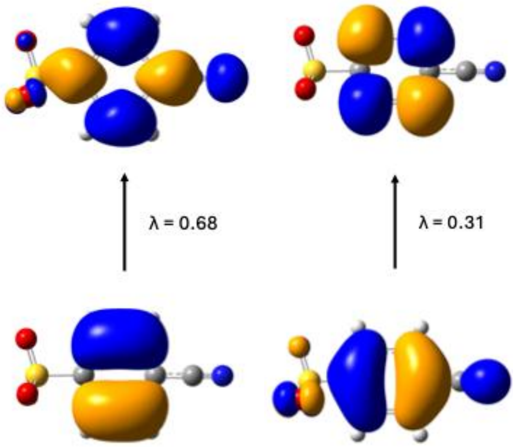
S0→S2 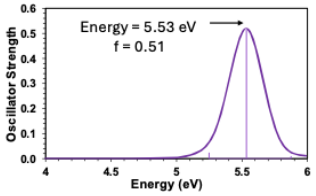	5.53	0.5166	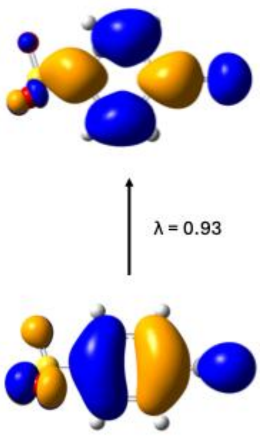

Table S5. Lowest Energy Transitions for 1d⁻ in MeCN from M06-2X/6-311+G(3df,2p) Calculations.

Transition	*E* / eV	*f*	NTOs
S0→S1 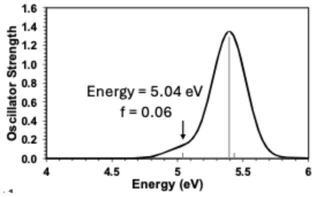	5.04	0.0550	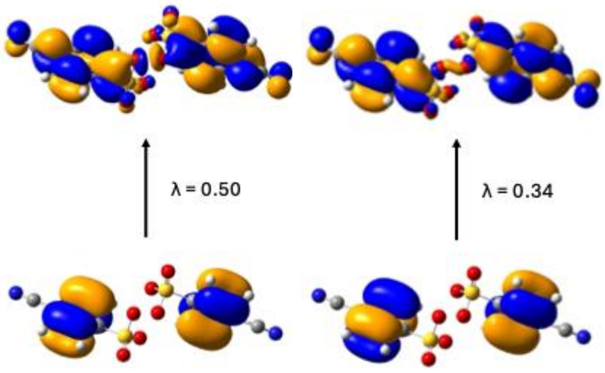
S0→S2 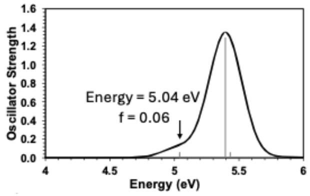	5.04	0.0553	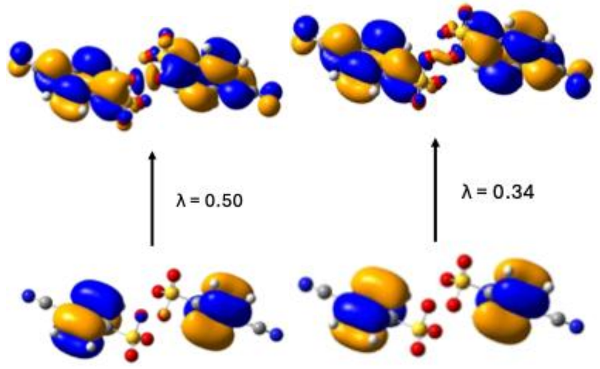
S0→S3 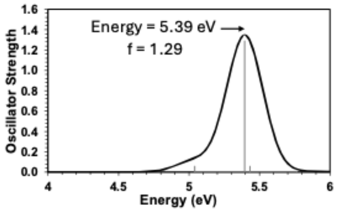	5.39	1.2889	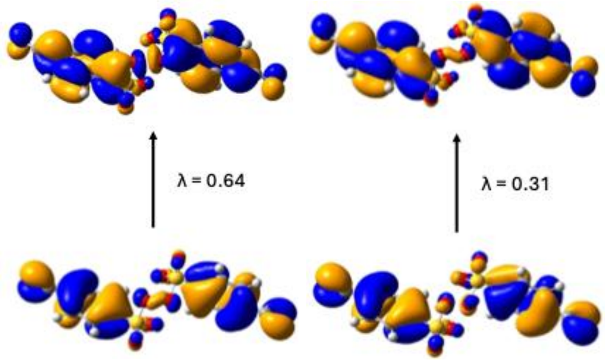
S0→S4 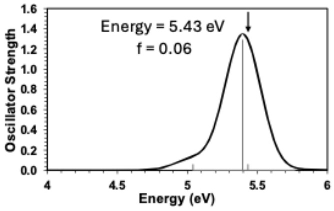	5.43	0.0581	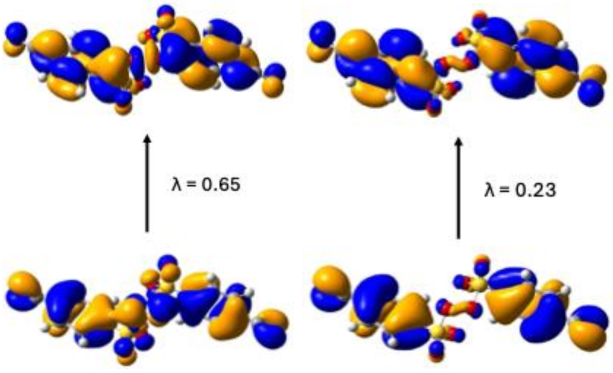

4. REFERENCES FOR SUPPORTING INFORMATION

(1) McCourt, R. O.; Studer, A. Bis(Arylsulfonyl) Peroxide-Mediated Difunctionalization of Cyclic Enol Ethers. *J. Org. Chem.* **2023**, *88*, 1860-1864.

(2) Mihai, M. T.; Williams, B. D.; Phipps, R. J. Para-Selective C–H Borylation of Common Arene Building Blocks Enabled by Ion-Pairing with a Bulky Countercation. *J. Am. Chem. Soc.* **2019**, *141*, 15477-15482.

(3) Keruckas, J.; Volyniuk, D.; Simokaitiene, J.; Narbutaitis, E.; Lazauskas, A.; Lee, P.-H.; Chiu, T.-L.; Lin, C.-F.; Arsenyan, P.; Lee, J.-H.; Grazulevicius, J. V. Methoxy- and *tert*-Butyl-Substituted *meta*-Bis(*N*-carbazolyl)phenylenes as Hosts for Organic Light-Emitting Diodes. *Org. Electron.* **2019**, *73*, 317-326.

(4) Farney, E. P.; Chapman, S. J.; Swords, W. B.; Torelli, M. D.; Hamers, R. J.; Yoon, T. P. Discovery and Elucidation of Counteranion Dependence in Photoredox Catalysis. *J. Am. Chem. Soc.* **2019**, *141*, 6385-6391.

(5) Puriņš, M.; Nakahara, H.; Levin, M. D. Bridging the Pyridine-Pyridazine Synthesis Gap by Skeletal Editing. *Science* **2025**, *389*, 295-298.

(6) Sohn, Y. S.; Hendrickson, D. N.; Gray, H. B. Electronic Structure of Metallocenes. *J. Am. Chem. Soc.* **1971**, *93*, 3603-3612.

(7) Wu, E. C.; Salamat, C. Z.; Ruiz, O. L.; Qu, T.; Kim, A.; Tolbert, S. H.; Schwartz, B. J. Counterion Control and the Spectral Signatures of Polarons, Coupled Polarons, and Bipolarons in Doped P3ht Films. *Adv. Funct. Mater.* **2023**, *33*, 2213652.

(8) Szabó, G.; Kamat, P. V. Spiro-Ometad: Unique Redox Chemistry Driving the Hole Transport. *ACS Energy Lett.* **2025**, *10*, 330-336.

(9) Roberts, J. G.; Voinov, M. A.; Schmidt, A. C.; Smirnova, T. I.; Sombers, L. A. The Hydroxyl Radical Is a Critical Intermediate in the Voltammetric Detection of Hydrogen Peroxide. *J. Am. Chem. Soc.* **2016**, *138*, 2516-2519.

(10) Chignell, C. F.; Motten, A. G.; Sik, R. H.; Parker, C. E.; Reszka, K. A Spin Trapping Study of the Photochemistry of 5,5-Dimethyl-1-Pyrroline *N*-Oxide (DMPO). *Photochem. Photobiol.* **1994**, *59*, 5-11.

(11) Misovich, M.; Folarin, H.; Paul, S. K.; Walter, R.; Everly, R. M.; Baltrusaitis, J.; Slipchenko, L. V.; Laskin, A. Electron Spin Resonance Study of Radicals Produced from Organic Triplet Excited States in Photolyzed Aqueous Mixtures. *J. Phys. Chem. A* **2025**, *129*, 4265-4274.
